# Gene Loss and Error-Prone RNA Editing in the Mitochondrion of *Perkinsela*, an Endosymbiotic Kinetoplastid

**DOI:** 10.1128/mBio.01498-15

**Published:** 2015-12-01

**Authors:** Vojtěch David, Pavel Flegontov, Evgeny Gerasimov, Goro Tanifuji, Hassan Hashimi, Maria D. Logacheva, Shinichiro Maruyama, Naoko T. Onodera, Michael W. Gray, John M. Archibald, Julius Lukeš

**Affiliations:** a Institute of Parasitology, Biology Centre, Czech Academy of Sciences, České Budějovice, Czech Republic; b Faculty of Sciences, University of South Bohemia, České Budějovice, Czech Republic; c Life Science Research Centre, Faculty of Science, University of Ostrava, Ostrava, Czech Republic; d Faculty of Biology, Lomonosov Moscow State University, Moscow, Russia; e Department of Biochemistry and Molecular Biology, Dalhousie University, Halifax, Canada; f Faculty of Bioengineering and Bioinformatics, Lomonosov Moscow State University, Moscow, Russia; g Canadian Institute for Advanced Research, Toronto, Canada; Max Planck Institute for Marine Microbiology

## Abstract

*Perkinsela* is an enigmatic early-branching kinetoplastid protist that lives as an obligate endosymbiont inside *Paramoeba* (Amoebozoa). We have sequenced the highly reduced mitochondrial genome of *Perkinsela*, which possesses only six protein-coding genes (*cox1*, *cox2*, *cox3*, *cob*, *atp6*, and *rps12*), despite the fact that the organelle itself contains more DNA than is present in either the host or endosymbiont nuclear genomes. An *in silico* analysis of two *Perkinsela* strains showed that mitochondrial RNA editing and processing machineries typical of kinetoplastid flagellates are generally conserved, and all mitochondrial transcripts undergo U-insertion/deletion editing. Canonical kinetoplastid mitochondrial ribosomes are also present. We have developed software tools for accurate and exhaustive mapping of transcriptome sequencing (RNA-seq) reads with extensive U-insertions/deletions, which allows detailed investigation of RNA editing via deep sequencing. With these methods, we show that up to 50% of reads for a given edited region contain errors of the editing system or, less likely, correspond to alternatively edited transcripts.

## IMPORTANCE

Uridine insertion/deletion-type RNA editing, which occurs in the mitochondrion of kinetoplastid protists, has been well-studied in the model parasite genera *Trypanosoma*, *Leishmania*, and *Crithidia*. *Perkinsela* provides a unique opportunity to broaden our knowledge of RNA editing machinery from an evolutionary perspective, as it represents the earliest kinetoplastid branch and is an obligatory endosymbiont with extensive reductive trends. Interestingly, up to 50% of mitochondrial transcripts in *Perkinsela* contain errors. Our study was complemented by use of newly developed software designed for accurate mapping of extensively edited RNA-seq reads obtained by deep sequencing.

## INTRODUCTION

Kinetoplastids are a diverse, widespread, and ecologically significant group of protists, some of which are devastating human parasites. Kinetoplastids have been the focus of intense research, mainly because of the medical importance of *Leishmania* and *Trypanosoma* species, and have been shown to exhibit a variety of unique cellular and molecular features, including RNA editing, mRNA *trans*-splicing, and genes arranged in polycistronic arrays (1). However, relatively little is known about the origin and evolution of these features across the full breadth of kinetoplastid diversity, despite the fact that there is tremendous species richness in both terrestrial, obligatory parasitic trypanosomatids (2) and free-living marine bodonids (3).

Insertion/deletion of uridine (U) residues (U-indel) into/from the mitochondrial mRNAs of kinetoplastids was the first type of RNA editing discovered (4). A plethora of posttranscriptional modifications have subsequently been described in organisms ranging from bacteria to plants and humans (for a review, see reference 5). RNA editing events include various insertions and deletions of single or multiple residues as well as base modifications and replacements, and they occur in both noncoding and protein-coding RNAs transcribed from nuclear and/or organellar genomes (6, 7). Numerous types of conversion editing have been implicated in a wide range of cellular processes, including embryonic development of the brain (8) and cancer (9).

While RNA editing in general seems to be particularly abundant in mitochondria and plastids (10, 11), U-indel RNA editing is at present confined to the mitochondria of kinetoplastids (1, 12, 13) and their sister clade, Diplonemea (14–16). U-indel editing is the most complex form of RNA editing known. Multiple sites within most transcripts are edited, with some mRNAs edited over their entire length (so-called pan-editing). In the model kinetoplastid *Trypanosoma brucei*, more than 70 different proteins have been shown to be incorporated into numerous dynamic editing complexes (12, 13), and up to 1,000 different small RNA molecules, called guide RNAs (gRNAs), act as the templates that define editing sites along a cognate mRNA (17).

Another unusual feature of kinetoplastid mitochondria is the structure and composition of their ribosomes. In *T. brucei*, 129 mitochondrial ribosomal proteins are encoded in the nucleus and targeted to the organelle posttranslationally (18). Only a single ribosomal protein, RPS12 (19), and two rRNAs are encoded in the mitochondrial genome. The bulk of the mitochondrial DNA (mtDNA) (or kinetoplastid DNA [kDNA]) of kinetoplastids is made up of minicircles encoding gRNA genes (13). The 9S and 12S mitochondrial rRNAs of *T. brucei* are highly truncated and lack several conserved domains that are functionally significant in other eukaryotes (20). Their transcription is developmentally regulated, and they are 3′-polyuridylylated (21). Determination of the high-resolution three-dimensional structure of a protein-rich, rRNA-poor mitochondrial ribosome of a related species, *Leishmania tarentolae*, was instrumental in explaining the shrunken mitochondrial rRNAs (22).

We are studying the molecular biology and evolution of the early-branching kinetoplastid *Perkinsela*. Members of this morphologically divergent, flagellum-lacking genus live as obligate endosymbionts inside marine amoebae (23), which to our knowledge is the only known example of a coevolving endosymbiotic relationship between two nonphotosynthetic eukaryotes. The kinetoplastid-amoeba symbiotic system appears to have emerged early in the evolution of the genus *Paramoeba* (24). The closest known relative of *Perkinsela* is the fish ectoparasite *Ichthyobodo necator*, and both of these kinetoplastids belonging to the Prokinetoplastina clade (25), currently represented by a relatively small number of species in rRNA databases (26). Within the confines of the host amoeba cytoplasm, *Perkinsela* is sometimes referred to as the “parasome” or “*Perkinsela*-like organism (PLO).” Amoeba hosts include free-living and facultatively parasitic marine amoebae of the genera *Paramoeba* and *Janickina* (24, 27–29). The *Perkinsela* strains studied here are associated with *Paramoeba pemaquidensis*, the causative agent of amoebic gill disease, which results in considerable mortality at marine fish farms (30, 31).

Using *Perkinsela* and *Paramoeba* genomic and transcriptomic data (GenBank accession number LFNC00000000), we have assembled the mitochondrial genomes of *Perkinsela* strains CCAP1560/4 and GillNOR1/I and characterized their overall structures and expression with particular attention to RNA editing. Furthermore, we have predicted the composition of their respiratory chain complexes, as well as the proteins involved in RNA editing, processing, and translation. We show that the mitochondrial genome of *Perkinsela*, which is composed of a huge number of fragments with terminal repeats, has undergone a considerable reduction in gene content and that all detected protein-coding transcripts undergo extensive U-indel RNA editing. While most proteins associated with RNA editing and with mitochondrial ribosomes in *T. brucei* are recognizable in *Perkinsela*, mitochondrial rRNAs were not found despite an exhaustive search, suggesting that they are fragmented and/or extremely divergent, similar to the situation observed in the related diplonemid *Diplonema papillatum* (16).

Importantly, we have conducted, to our knowledge, the first investigation of U-indel-edited mitochondrial transcripts based on deep transcriptome sequencing (RNA-seq), and we have developed software tools for accurate mapping of extensively edited reads. Since the discovery of this type of RNA editing in 1986, editing mechanisms have been unraveled via targeted sequencing on a clone-by-clone basis (32–34). Recently, deep sequencing of gRNA libraries in *T. brucei* (17, 35) has uncovered an unexpected degree of complexity and disorder inherent in gRNA-mediated editing. By deep sequencing of mRNAs, we have unveiled an even greater level of complexity in the form of “misediting” (36–38), although we have not detected alternative translatable mRNAs with considerable abundance.

## RESULTS AND DISCUSSION

### *Perkinsela* mitochondrial genome structure.

**FIG 1:**
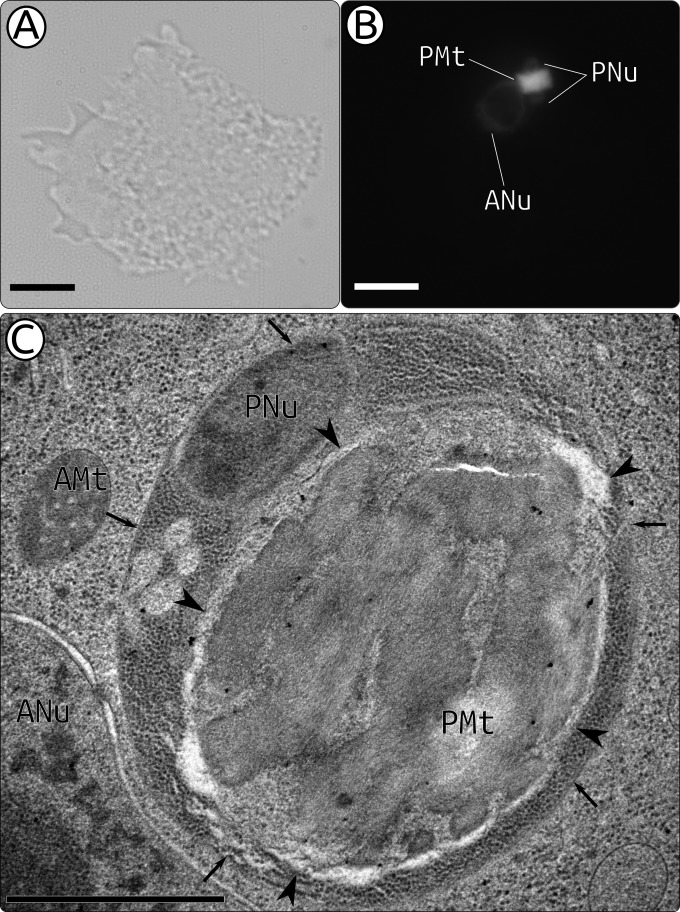
Phase-contrast image (A), DAPI staining image (B), and high-pressure freezing transmission electron microscopy image (C) of *Perkinsela* strain GillNOR1/I. Scale bars, 10 µm (A and B) or 1 µm (C). In panel C, small arrows mark the single membrane separating *Perkinsela* from the amoeba host cytoplasm, and arrowheads mark the outer mitochondrial membrane of *Perkinsela*. Abbreviations: ANu, amoeba nucleus; PNu, *Perkinsela* nucleus; PMt, *Perkinsela* mitochondrion.

*Perkinsela* can be visualized in the *Paramoeba* cell (Fig. 1A) via 4′,6-diamidino-2-phenylindole (DAPI) staining of DNA, which shows that the endosymbiont is invariably located in the perinuclear region of amoebae (Fig. 1B and C). Interestingly, based on the intensity of DAPI staining, it appears that *Perkinsela* harbors a larger amount of DNA in its mitochondrion (kDNA) than in the rather inconspicuously stained nuclei of *Perkinsela* and *Paramoeba* (Fig. 1B). High-pressure freezing transmission electron microscopy, which optimally preserves the fine structure, confirmed an earlier observation obtained by standard electron microscopy (23, 39), namely, that the single mitochondrion of *Perkinsela* is packed with kDNA strands arranged in parallel electron-dense layers (Fig. 1C). This observation, which is further supported by the fact that it is impossible to arrange *Perkinsela* mitochondrial contigs on one scaffold by using mate-pair reads (data not shown), suggests that the kDNA of *Perkinsela* is not composed of minicircles and maxicircles, as is the case with the genera *Trypanosoma* and *Leishmania*, and other trypanosomatids. Molecules constituting the kDNA of bodonids studied thus far do not fall into distinct categories of mini-circles and maxi-circles, and they also do not form a single catenated kDNA network (40). Both features also seem to be present in *Perkinsela*, an early-branching bodonid (27). Moreover, since both DAPI staining and electron microscopy show that the kDNA and the single mitochondrion occupy most of the *Perkinsela* cell volume and that the organellar genome constitutes the most abundant DNA in this endosymbiont-host system, it is likely that this inflated genome is present at an extremely high copy number.

**FIG 2:**
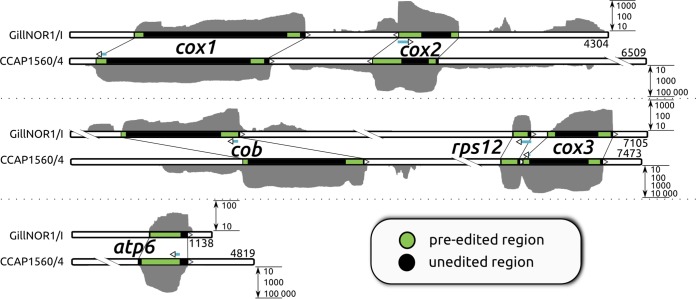
Gene-bearing mitochondrial scaffolds identified in *Perkinsela* strains CCAP1560/4 (GenBank accession numbers KT261384 to KT261386) and GillNOR1/I (GenBank accession numbers KT272167 to KT272169). Transcript regions undergoing RNA editing are shown in green. Scaffold 1 contains *cox1* and *cox2* genes in reverse orientation; scaffold 2 contains *cob* and closely spaced *rps12* and *cox3* genes in the same orientation; scaffold 3 contains only the *atp6* gene. While most transcripts are edited in separate regions at their ends, *rps12* and *atp6* are edited over almost their entire lengths, i.e., pan-edited. Gene regions used for detailed mapping of alternatively edited reads (see File S1 in the supplemental material) are shown by teal arrows, which also indicate the direction of RNA editing in these regions. For the GillNOR1/I strain, coverage with strand-specific RNA-seq reads (with U-indel optimized settings) for each transcript is shown in the sense orientation only (for both the sense and antisense reads that were plotted, see Fig. S1 in the supplemental material). For CCAP1560/4, RNA-seq reads were not strand-specific. Coverage (gray blocks) is plotted on a logarithmic scale. Absolute values of coverage were markedly different for the two strains due to the different sequencing approaches used (see File S2 in the supplemental material).

Trypanosomatid mtDNAs invariably have a complement of 18 protein-coding genes and two rRNA genes (1). However, individual flagellate species differ in gene regions at which posttranscriptional U-indel editing takes place (41, 42). Out of this conserved gene set, we identified just six protein-coding genes (*cox1*, *cox2*, *cox3*, *cob*, *atp6*, and *rps12*) on three assembled mitochondrial contigs in *Perkinsela* that were similar in both studied strains (Fig. 2). Unlike typical trypanosomatid maxicircles, which have a single variable region and a dense protein-coding region (43), these *Perkinsela* contigs contain short tandem repeats at their ends. No similarity was found among repetitive sequences flanking the three protein-coding contigs (data not shown). Unambiguous connection of the contigs into larger scaffolds proved impossible, despite the usage of mate-pair reads for scaffolding and despite manual analysis of a contig graph produced using the assembly software GS *de novo* Assembler v.2.9 (Newbler).

**FIG 3:**
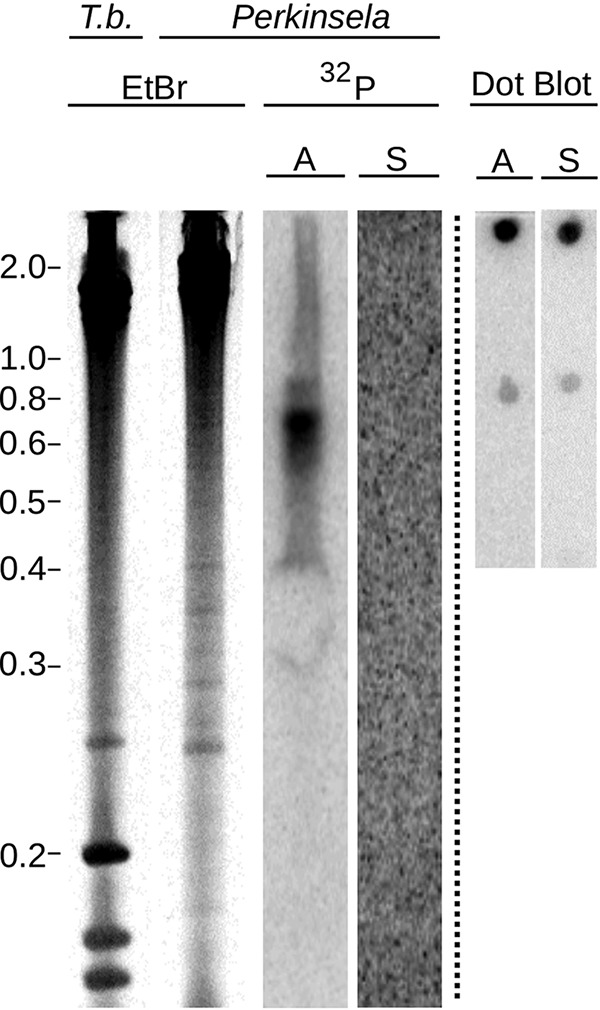
Northern blot with antisense and sense probes for the *cox2* transcript, showing that only a single strand of the *cox2* gene is transcribed. Total RNA from *T. brucei* (*T.b.*) and *Perkinsela* resolved on a denaturing gel was visualized via ethidium bromide (EtBr) stain. The signal from the antisense *cox2* probe is shown in the lane labeled “^32^P, A,” while the sense-probed Northern membrane is shown in the lane labeled “^32^P, S.” Dot blots simultaneously probed with antisense (A) and sense (S) probes are shown on the right, with increasing dilutions from top to bottom of a denatured plasmid bearing an insert corresponding to the probed sequence.

This highly reduced set of a half-dozen genes encodes subunits of three respiratory complexes: *cob* of complex III (ubiquinone-cytochrome *c* oxidoreductase); *cox1*, *cox2*, and *cox3* of complex IV (cytochrome *c* oxidase); and *atp6* of complex V (ATP synthase), suggesting a functional respiratory chain. The apparent absence of respiratory complex I in *Perkinsela* (in which mtDNA-encoded subunits *nad1* through *nad9* are missing) is further supported by the absence of the nucleus-encoded subunits of this complex (see below; see also Table S1 in the supplemental material). All six mtDNA protein-coding genes are transcribed (with varied transcript abundance levels) and undergo U-indel editing to slightly different degrees (Fig. 2; Table 1). Long antisense transcripts were undetectable by Northern blotting, at least in the case of *cox2* (Fig. 3). Due to the extremely slow growth of *Paramoeba* in culture, we were not able to accumulate enough RNA for testing antisense transcription of other *Perkinsela* mitochondrial genes by Northern blotting, but mapping of strand-specific RNA-seq reads revealed no significant antisense transcripts in strain GillNOR1/I (see Fig. S1 in the supplemental material).

**TABLE 1:** Statistics for edited mitochondrial mRNAs in *Perkinsela*^a^

Strain	Gene	Pre-edited size (nt)	Edited size (nt)	% size increase	% U in ORF	Protein length (aa)	Pre-edited region(s) length^b^ (nt)	U insertions^b^	U deletions^b^	No. of edited sites^b^
CCAP1560/4	*cob*	964	1,136	18	45	370	35 + 134	41 + 146	3 + 13	14 + 68
GillNOR1/I		936	1,125	20	47	370	36 + 134	45 + 141	4 + 8	15 + 66
CCAP1560/4	*cox1*	1,374	1,567	14	40	521	78 + 107	77 + 121	3 + 3	31 + 54
GillNOR1/I		1,365	1,589	16	42	521	68 + 103	77 + 125	2 + 3	30 + 53
CCAP1560/4	*cox2*	486	661	36	51	209	55 + 227	57 + 151	6 + 28	23 + 102
GillNOR1/I		487	710	46	49	209	57 + 177	58 + 148	8 + 26	24 + 98
CCAP1560/4	*cox3*	656	814	24	44	256	45 + 89	69 + 93	2 + 2	31 + 47
GillNOR1/I		624	801	28	49	255	54 + 97	71 + 93	3 + 2	31 + 47
CCAP1560/4	*rps12^c^*	157	268	72	55	80	123	123	13	52
GillNOR1/I		150	257	72	55	80	110	123	12	52
CCAP1560/4	*atp6^c^*	363	651	80	61	197	309	318	30	154
GillNOR1/I		321	625	94	62	197	298	311	28	152

a Only the main edited products were taken into account.

b Numbers correspond to the 5′ and 3′ regions, respectively.

c Pan-edited transcript, i.e., edited throughout most of its length.

Despite extensive searching, rRNA genes could not be identified by BLAST using known kinetoplastid and *Diplonema* rRNA genes as queries. Further candidate sequences were obtained from transcribed regions of the assembly not assigned to the *Perkinsela* or host nuclear genomes. In addition, *Perkinsela* reads containing the peptidyl transferase core motif ACCTCGNTGT conserved even in the highly diverged *Diplonema* large subunit (LSU) rRNA (16) were assembled, and the resulting contigs were added to the list of putative rRNA-encoding sequences. All top candidates were subjected to a careful manual secondary structure prediction with the help of the Mfold software (terminal hairpin prediction), but no small subunit (SSU) or LSU rRNA-like folds were found (data not shown).

In light of the recent discovery of a split and edited LSU rRNA in *Diplonema*, a relative of *Perkinsela*, and the fact that the SSU rRNA of *Diplonema* remains unidentified (16), it seems likely that extreme divergence and/or fragmentation renders the mitochondrial rRNAs of *Perkinsela* unrecognizable. We consider it highly improbable that the mitochondrial rRNA is genuinely absent, because upon RNA editing, detected transcripts have evolutionarily conserved open reading frames, implying the requirement of a functional ribosome to translate them into protein. Moreover, both universal and kinetoplastid-specific mitochondrial ribosomal proteins are generally conserved in *Perkinsela* (see Table S1 in the supplemental material), and a ribosomal subunit gene (*rps12*) is also present in its organellar genome (Fig. 2).

We searched for putative gRNA genes in the genome assembly of *Perkinsela* strain CCAP1560/4 that had the following characteristics: (i) at least 25 nucleotides (nt) complementary to the final edited transcript, (ii) up to 5 G-U pairs allowed, and (iii) supported by at least one transcriptomic read. Using this approach, we pinpointed 65 candidate gRNA genes in 29 contigs, matching all protein-coding transcripts except for *rps12* (see File S3 in the supplemental material). The average length of matching contigs was 730 bp (from 176 to 3,021 bp). Flanking regions of putative gRNA genes had no common motifs, except for an A- or an A/C-rich tract upstream of the mRNA-complementary region (see File S3). However, we could not obtain robust gRNA candidates for two reasons. First, a dedicated library of short transcripts was lacking, and any standard RNA-seq library is depleted in short transcripts. Second, a strand-specific library is necessary to prove that putative gRNA genes produce transcripts which are antisense relative to the protein gene transcript; however, the strand-specific RNA-seq library we constructed for *Perkinsela* strain GillNOR1/I had relatively low coverage of mitochondrial transcripts and produced few gRNA candidates (data not shown).

### Nucleus- and mitochondrion-encoded respiratory chain subunits.

**FIG 4:**
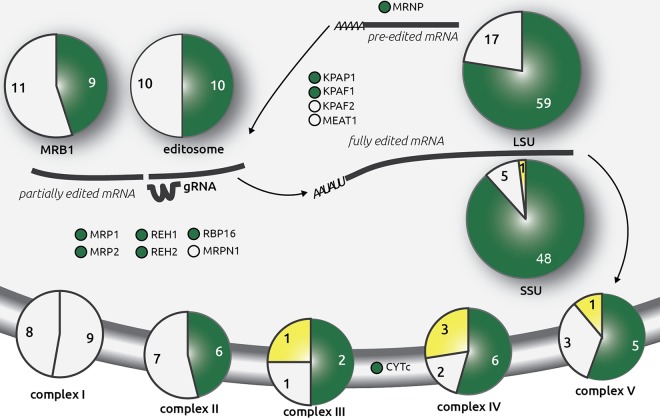
Conservation of respiratory chain subunits, RNA editing and processing factors, and mitochondrial ribosomal proteins in *Perkinsela*. Each complex is represented as a pie chart, and numbers indicate subunits analyzed in this study. The green color marks proteins identified in the *Perkinsela* genome (also listed in Table S1 in the supplemental material). Missing proteins are shown in white, and proteins encoded in the mitochondrial genome are shown as bright yellow areas. The left-hand section of the pie chart for respiratory chain complex I represents subunits encoded in the mitochondrial genomes of trypanosomatids but missing in *Perkinsela*. The following complexes are shown: respiratory chain complexes I to V, the RECC (editosome), mitochondrial RNA-binding complex 1 (MRB1), and the LSU and SSU of the mitochondrial ribosome. A number of other proteins involved in mRNA/gRNA processing are also shown (see Table S1 for definitions of protein acronyms).

Using hidden Markov models (HMM) constructed on the basis of trypanosomatid orthologs, the *Perkinsela* genomic contigs (GenBank accession number LFNC00000000) were searched for mitochondrial proteins (see Materials and Methods for details). Since none of the nucleus-encoded subunits of the respiratory complex I (NADH dehydrogenase) were detected, we considered this component of the respiratory chain missing in *Perkinsela* (Fig. 4; see also Table S1 in the supplemental material). This inference is in agreement with our failure to detect any of the mtDNA-encoded subunits of complex I in the mitochondrial contigs. Similarly, no alternative NADH dehydrogenases were found. The other respiratory complexes (II through V) that together mediate oxidative phosphorylation are apparently present in *Perkinsela* (Fig. 4; see also Table S1). We conclude that in the mitochondrion of *Perkinsela* the respiratory chain is functional, with the missing complex I likely replaced by an as-yet-unidentified alternative NADH dehydrogenase. Although the distantly related *T. brucei* possesses both mitochondrion- and nucleus-encoded subunits of complex I, its function remains elusive, with a highly active alternative dehydrogenase substituting for the canonical biochemical activity (44, 45). It thus seems that in kinetoplastids, complex I is prone to loss and was eliminated in the early-branching *Perkinsela*.

### RNA editing and processing complexes, mitochondrial ribosomes.

We next verified the presence of nucleus-located genes for proteins imported into the *Perkinsela* mitochondrion by using *T. brucei* as a reference. Despite its endosymbiotic lifestyle and large evolutionary distance from other kinetoplastid flagellates, *Perkinsela* has generally conserved kinetoplastid mitochondrial transcription and translation machineries, as well as a complex RNA editing machinery (Fig. 4; see also Table S1 in the supplemental material). The composition of these protein complexes is described below.

Transcription of the mitochondrial genome is performed by a dedicated single-subunit, phage T3/T7-like RNA polymerase (46) which is present in *Perkinsela*. In trypanosomatids, the formation of short A-tails on pre-edited mRNAs and long A/U tails on fully edited transcripts is controlled by kinetoplast poly(A) polymerase 1 (KPAP1), 3′-terminal uridylyl transferase (TUTase) KRET1, and their accessory factors, KPAF1 and KPAF2, which together regulate mRNA translatability and stability (47). Except for KPAF2, all these nucleus-encoded and mitochondrion-targeted proteins are present and well conserved in *Perkinsela*. KRET1 also appends 3′-oligo(U) tails to rRNAs and gRNAs in trypanosomatids (48), so it seems reasonable to assume that this enzyme performs the same function in *Perkinsela*.

The core set of editing reactions in trypanosomatid mitochondria is executed by the RNA editing core complex (RECC), also called the 20S editosome (13). In the first step of the editing reaction, the cleavage of the mRNA at a mismatch between it and a hybridizing gRNA yields 5′ and 3′ fragments bridged by the gRNA, and this reaction is performed by one of three RECC endonucleases (49). Remarkably, among these three endonucleases, only a homolog of the U-insertion-specific enzyme KREN2 was found in *Perkinsela*; KREN1 (the deletion-specific endonuclease) and KREN3 were not detected. KREN3 is known to act on the *cox2* transcript edited by a *cis*-gRNA located in its 3′ untranslated region in trypanosomatids (50). Of the KREPB proteins (KREPB6 through 8), which within RECC form dimers with the KREN endonucleases (51), only KREPB6, which in *T. brucei* interacts with KREN3, was found. KREPB8 and KREPB7, which dimerize with KREN1 and KREN2, respectively, are apparently absent in *Perkinsela*. With regard to the deletion of extraneous Us from the 5′ mRNA fragment (52), the dedicated exonucleases KREX1 and KREX2 have predicted orthologs in *Perkinsela*. The KRET2 TUTase, responsible for adding Us to the 5′ mRNA fragment, and the insertion-specific RNA ligase KREL2, which reseals the two RNA fragments, were also found (53, 54). The deletion-specific RNA ligase KREL1 is missing in *Perkinsela*. Of the accessory and structural RECC subunits (KREPA1 through 6 and KREPB4 and 5), three are present, whereas five seem to be missing (see Table S1 in the supplemental material). The undetected orthologs were presumably replaced or have evolved beyond recognition in *Perkinsela*, or they are normally essential for editing transcripts encoding the numerous complex I subunits, which have been lost in this kinetoplastid.

In addition to the RECC, which provides the core editing enzymatic activities, various other proteins and macromolecular complexes have been shown to play vital roles in editing. One example is mitochondrial RNA-binding complex 1 (MRB1), a dynamic structure that binds and recruits gRNAs into the editing complex, processes massively edited mRNAs that require several gRNAs, and links RNA editing with mRNA tailing and translation machineries (12). Of six invariably recovered MRB1 subunits (55–58), three are found in *Perkinsela*, including the crucial gRNA-binding subunit GAP1, which presumably forms a homotetramer, as its paralog GAP2 is missing. Core subunits MRB5390 and MRB8620 are also absent from *Perkinsela* (see Table S1 in the supplemental material). Of 14 other putative editing complex members, only 5 are found, whereas TbRGG1, TbRGG2, MRB8170, and MRB4160 (58, 59) are missing in *Perkinsela* (see Table S1). However, TbRGG3, which associates with MRB1 as well as other mitochondrial RNA-binding proteins (60), yields a hit. Hence, the same picture emerges as for the 20S editosome: the functional core of the MRB1 complex is mostly conserved between *Perkinsela* and its trypanosomatid relatives.

A separate small complex, a heterotetramer of RNA-binding proteins 1 and 2 (MRP1/MRP2) which stimulates annealing of gRNA and mRNA molecules (61, 62), is also present in *Perkinsela*. The same is true for RNA-binding protein 16 (RBP16), which interacts with both mRNA and gRNA and has a multifunctional role in mitochondrial RNA metabolism (63). However, RNA processing endonuclease mRPN1, which is involved in cleavage of long gRNA precursor transcripts (64), was not detected, suggesting that gRNA transcription patterns may profoundly differ between trypanosomatids and *Perkinsela* (we did not attempt to identify gRNA genes in the latter). Finally, both RNA-editing helicases KREH1 and KREH2, likely required for unwinding the gRNA:mRNA duplex (55, 65, 66), have been detected in *Perkinsela*.

Ribosomes in trypanosomatid mitochondria contain extremely reduced rRNAs and have acquired a multitude of novel proteins, apparently to compensate for the loss of RNA domains (22), or through protein “accretion” by a neutral evolutionary mechanism (67). Thus, both ribosomal LSU and SSU contain dozens of trypanosomatid-specific proteins, but they lack some of the universally conserved ones (18). Of 27 mitochondrial LSU proteins conserved throughout eukaryotes, 25 are found in *Perkinsela* (see Table S1 in the supplemental material). Of 49 trypanosomatid-specific mitochondrial LSU subunits, only 15 could not be detected in *Perkinsela* (see Table S1). This significant conservation between *Perkinsela* and trypanosomatids is also seen for the SSU ribosomal proteins: in the case of 10 subunits universally present in mitochondrial ribosomes, only 1 is missing, whereas just 4 out of 43 trypanosomatid-specific proteins could not be detected in *Perkinsela* (see Table S1).

In summary, proteins incorporated into RNA editing and processing as well as translation machineries are generally conserved in *Perkinsela*, despite its deep evolutionary separation from *T. brucei* and other trypanosomatids (26).

### Analysis of edited RNA molecules.

We carried out an in-depth analysis of RNA editing based on thousands of Illumina reads per gene for *Perkinsela* strain CCAP1560/4, greatly surpassing the limits of traditional methods. We also took advantage of lower coverage but longer read sequence data (up to 450 bp long) generated for the GillNOR1/I strain (see File S2 in the supplemental material). Preliminary analyses revealed that read mapping with such a high fraction of U-indels is problematic, as publicly available read mapping software was not designed for such applications. Our initial approach using the Bowtie2 v.2.0.2 mapper with low indel penalties resulted in alignments that required extensive manual improvement due to misalignments in regions with closely spaced U-indel sites (data not shown). In order to improve mapping of U-indel-rich reads, we modified the Bowtie2 v.2.0.2 software, introducing nucleotide-specific gap opening and gap extension penalties into the Smith-Waterman alignment module (see Materials and Methods). Mapping reads with strict penalties for gaps containing A, C, or G but with relaxed penalties for gaps containing only U dramatically reduced the number of misalignments and improved the yield of edited reads (see Fig. S2 in the supplemental material). In the case of pan-edited transcripts or long editing domains, extra runs of mapping on partially edited templates were necessary to reconstruct the final edited product, as reads edited over the entire length lacked seeds long enough for initial mapping.

To overcome the problem of missing seeds, we developed a novel read mapping tool, T-aligner, based on the Smith-Waterman algorithm and designed to mimic the 3′-to-5′ progression of RNA editing in kinetoplastids. Initially, a fixed seed is chosen in a never-edited or universally edited 3′ terminus of the transcript (or editing domain, in appropriate cases), and then reads are mapped and the final edited sequence is reconstructed with the help of T-aligner (see Materials and Methods). At this stage, further iterations of read mapping are possible, shifting the seed in the 5′ direction. Using T-aligner, we identified mature edited transcripts in *Perkinsela* and investigated the extent to which alternative editing occurs.

RNA editing in *Perkinsela* resembles the system described in the model *Trypanosoma* and *Leishmania* species. However, the general distribution of editing sites (Fig. 2; Table 1), namely, the fact that the 3′ and 5′ regions of genes usually contain separate editing domains, more closely resembles the situation in the bodonid *Trypanoplasma borreli*, for which only a few genes and transcripts have been sequenced (41). Interestingly, in the case of the *Perkinsela cox2* gene, we found that the 5′ domain is edited prior to the 3′ domain, despite the canonical 3′-to-5′ progression of U-indel editing inside these domains (33). Upon inspection of the longest read fraction, we observed no reads in which the 3′ domain was at least partially edited when the 5′ domain was not (see Fig. S3 in the supplemental material).

**FIG 5:**
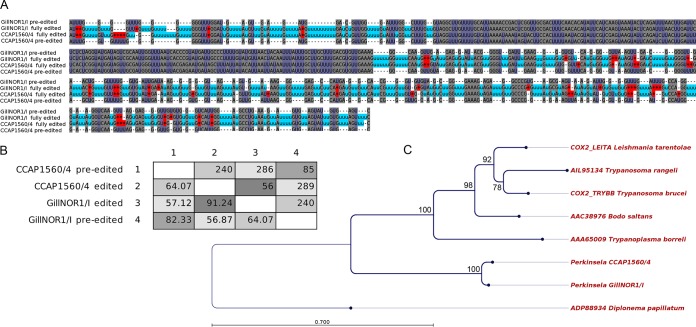
U-indel editing in the *cox2* mRNA of *Perkinsela* strains CCAP1560/4 and GillNOR1/I. (A) Alignment of edited and pre-edited transcript sequences. U insertions and deletions are highlighted in light blue and red, respectively. (B) Pairwise percent identities (in the lower left half of the matrix) and numbers of different positions (upper right) between edited/pre-edited sequences of both strains. (C) A maximum likelihood unrooted tree of COX2 proteins of *Perkinsela* and other kinetoplastids, and of *Diplonema papillatum*, used as an outgroup. The tree was constructed with the following settings: WAG+Г substitution model, neighbor-joining starting tree, 1,000 bootstrap replicates. Branches supported by bootstrap values of >70% are shown with thicker lines. Scale bar shows the inferred number of amino acid substitutions per site.

In total, fully edited versions of six transcripts have 1,196 Us inserted and 103 Us deleted at 576 distinct edited sites in the *Perkinsela* CCAP1560/4 strain, and there are 1,192 Us inserted and 92 Us deleted at 568 edited sites in the GillNOR1/I strain (Table 1). Alignments of edited and preedited mRNAs, their translation, and the trees built for predicted proteins and their kinetoplastid orthologs are shown for *cox2* in Fig. 5 (and for the other five mitochondrial genes in File S4 of the supplemental material). Finding a protein with an expected length and an expected position in a phylogenetic tree constitutes strong *in silico* evidence that the predicted translation product from a reconstructed edited mRNA sequence is most probably correct. The divergence of editing patterns between the two studied isolates (Fig. 5A and B; see also File S5 in the supplemental material) is similar to that observed among various species of trypanosomatids (68), and sequences become noticeably less divergent following RNA editing, as shown in Fig. 5B for *cox2*: preedited mRNA sequences of the two *Perkinsela* strains have 82% identity, while the respective edited molecules have 91% identity (85 and 56 nucleotide differences, respectively). Indeed, this effect is even more pronounced in the case of the pan-edited *atp6* transcript, with just 79% identity of preedited mRNAs between the strains but with 94% identity after posttranscriptional modification (see File S4). These results are consistent with the notion that while protein sequences are maintained by selective forces, the sequence of a cryptic gene is able to evolve more freely, with mutations “corrected” by RNA editing (68).

### Alternative editing and “misediting.”

We observed a certain fraction of alternatively edited reads for each of the 10 edited transcript domains in the *Perkinsela* mitochondrial genome. We defined “alternative” reads as those containing at least one alternatively edited site that satisfied the following conditions: (i) it was never edited in the main editing product; (ii) the U-indel was longer than in the main product; and (iii) insertion occurred instead of deletion in the main product, or vice versa. Short alternatively edited reads fully contained within longer reads were not considered in further analyses. The fraction of alternatively edited reads (relative to all edited reads), as inferred using T-aligner, was found to vary from 19% in the investigated edited domains of *cox2*, *cox3*, and *cob* to 52% in the pan-edited *atp6* transcript (see File S5 in the supplemental material). Absolute numbers of alternative reads in our data set varied from 44 for *rps12* to 1,979 for *atp6*, depending on the level of coverage for a particular strain, transcript abundance, and T-aligner seed selection (see File S5).

**FIG 6:**
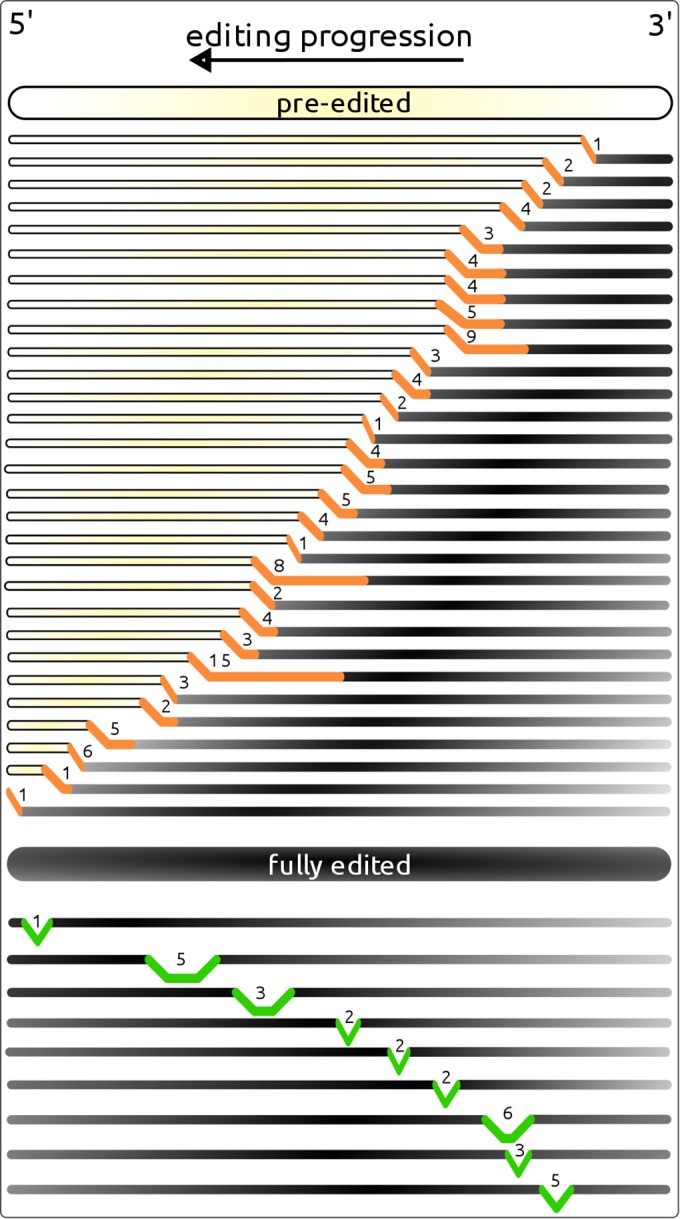
The most abundant alternatively edited intermediates mapped to the *cox2* transcript (3′ edited domain) in *Perkinsela* strain GillNOR1/I. The preedited sequence is shown in yellow, and the main edited product is shown in black. Alternatively edited read fragments are shown in orange if they follow the sequence edited in a standard way or in green if they occur in the middle of such a sequence. The number of alternatively edited sites is shown in each case, and the length of highlighted regions correlates with the number of inserted Us.

Importantly, we observed no cases of a clearly predominant single alternative editing intermediate, and an overwhelming majority of alternative intermediates was represented by single reads, as shown in File S5 in the supplemental material. We used the following edited domains as model cases: (i) *cox1*, 5′ domain, strain CCAP1560/4; (ii) *cox2*, 3′ domain, strain GillNOR1/I; (iii) *cox3*, 5′ domain, strain CCAP1560/4; (iv) *cob*, 3′ domain, strain GillNOR1/I; (v) the 3′ portion of the pan-edited *atp6* transcript, strain CCAP1560/4; and (vi) the pan-edited *rps12* transcript, strain GillNOR1/I (see File S1 in the supplemental material). The *cox2* 3′ domain (Fig. 2) was most informative due to its high coverage with long strand-specific reads (average length, 192 nt; maximum length, ~400 nt; 4,711 edited reads in total) (see File S5 in the supplemental material). A maximum number of 14 alternatively edited sites was observed in the reads available for the 3′ domain of *cox2*. However, just 10 out of 880 reads mapping to this domain contained 10 or more alternatively edited sites (see File S5). An even larger pool of 1,979 alternatively edited reads mapping to the 3′ part of *atp6* contained just 6 reads with 10 or more alternatively edited sites. However, shorter reads were available in this case (see File S5). Taken together, these numbers, the read length distribution (see File S2 in the supplemental material), and the read counts for alternative intermediates (see File S5 in the supplemental material) strongly indicate that alternative final transcripts, comparable with the main transcript in length and abundance, do not occur in this system. A typical selection of alternative intermediates is shown for the 3′ edited domain of *cox2* (Fig. 6).

The majority of editing intermediates contained one or several alternative sites at the end of an edited stretch of sequence, followed by a preedited sequence. Considering that in *T. brucei* approximately 45 nucleotides (from nt 24 to 61) are covered by an average gRNA (17), the terminal stretches observed in *Perkinsela* are probably generated by one or two consecutively acting gRNAs. The paucity of longer terminal stretches (see File S1 in the supplemental material) suggests that we are mainly observing instances of abortive “misediting” (36). As is apparent even from a small selection of alternative intermediates (Fig. 6), editing errors occur almost everywhere along the transcript. However, a wider selection of intermediates (see File S1) revealed a few hot spots.

Another type of editing intermediate contains one to six alternative sites within a sequence corresponding to the main editing pathway. These “internal” intermediates are apparently produced by a single gRNA guiding several editing sites in a noncanonical way, but still generating an anchor sequence for a subsequent gRNA in the main editing pathway. Remarkably, both types of alternative editing have been predicted in *T. brucei* based on deep sequencing of a gRNA library with a total of ~600 major sequence classes (17); gRNAs were identified that create an alternative sequence not usable as an anchor, as were gRNAs that edit several sites in an alternative way but create an anchor region for the next gRNA in the main editing pathway. For instance, an alternative gRNA might initiate editing at the 3′ end of *atp6* (also known as A6) in *T. brucei* but is also able to create a normal anchor for the next gRNA. The same is true for alternative gRNA editing of the NAD8 transcript (*nad8*). In *Perkinsela*, we also observed intermediates containing more than one internal alternatively edited stretch, or intermediates with a combination of terminal and internal alternatively edited stretches (see File S1 in the supplemental material), all of which are of low abundance.

Based on our data, the RNA editing pathway in *Perkinsela* and probably all kinetoplastids can be viewed as a “tree” with numerous branching points, with only one path in the tree being predominant and the rest probably representing errors of the editing system. In *T. brucei*, alternative gRNAs were identified for at least five genes, with some being even more abundant than the standard gRNAs for the same site (17). Given a high percentage of alternative reads accumulated for some edited domains in *Perkinsela* (e.g., 52% for *atp6*), and the fact that alternative reads that map to the *rps12* gene lack stop codons in at least one frame, the mitochondrial transcription-translation system in this organism, in one way or another, has to cope with a large number of “incorrect” transcripts. In plant organelles, only edited translation products appear to accumulate in mitochondrial ribosomes (69). Whether the *Perkinsela* mitochondrion is able to tolerate “incorrect” protein products, or that some sort of discrimination by the translation machinery is in place, remains an open question.

## MATERIALS AND METHODS

### Cell culture.

*Paramoeba pemaquidensis* strain CCAP1560/4 was obtained from the CCAP (Culture Collection of Algae and Protozoa). Cells were grown on MYS medium (0.01% malt extract and 0.01% yeast extract in artificial seawater, solidified with 1.5% agar) (70). *P. pemaquidensis* strain GillNOR1/l was obtained from the culture collection of the Institute of Parasitology, Czech Academy of Sciences, and was grown on MY75S medium (0.01% malt extract and 0.01% yeast extract in artificial seawater, solidified with 2.0% agar). Both strains were grown in the dark at 20°C, and cells were collected after about 3 months.

### Microscopy.

*P. pemaquidensis* strain GillNOR1/I, carrying *Perkinsela* and feeding on diverse bacteria, was grown on agar plates, and the cells were collected as described previously (23). Cells were prepared for phase-contrast microscropy, DAPI epifluorescence light microscopy, and high-pressure freezing transmission electron microscopy following protocols described elsewhere (71, 72).

### *Paramoeba pemaquidensis* sequencing.

Two strains of *P. pemaquidensis* with their respective *Perkinsela* endosymbionts were used in this study. Strains CCAP1560/4 (73) and GillNOR1/I were isolated from gills of Atlantic salmon captured in the waters of Wales and Tasmania, respectively (74). We prepared and sequenced total genomic and poly(A)-enriched transcriptomic libraries from strain GillNOR1/I (see File S2 in the supplemental material) and genomic, poly(A)-enriched transcriptomic and spliced leader RNA-enriched transcriptomic libraries for strain CCAP1560/4. The latter library was used for assigning contigs to the *Perkinsela* nuclear genome, as it is currently impossible to physically separate *Perkinsela* from its amoeba host, or to separate their DNAs (GenBank accession number LFNC00000000), and mRNAs “capped” with a spliced leader RNA do not occur in the amoeba.

### Mitochondrial genome assembly.

Raw DNA sequence reads from all sequencing platforms were filtered and trimmed to ensure quality and then depleted of adapter sequences, and paired-end transcriptomic reads were merged using the CLC Genomics Workbench v.6.5 (see File S2 in the supplemental material). The mitochondrial genomes of both *Perkinsela* strains were assembled from combined next-generation sequencing reads with the Newbler Assembler (GS *de novo* Assembler v.2.9), from single 454, mate-pair, and paired-end Illumina HiSeq reads in the case of strain CCAP1560/4 and from paired-end Illumina MiSeq reads for strain GillNOR1/I (see File S2). A number of assembly parameters were tested with the goal of maximizing mitochondrial contig size. Manual analysis of a graph of alternative contig connections (produced by Newbler) with an in-house visualizing script was used to close gaps and assemble long repetitive regions. RNA-seq assemblies were performed with Trinity software (75).

### Gene identification.

Proteins predicted from the *Perkinsela* mitochondrial contigs and translated transcriptomic assemblies were initially identified using the HMMER3 software. Available kinetoplastid and diplonemid mitochondrial protein sequences were used for the construction of hidden Markov models (HMMs), which were subsequently used as queries against conceptual translations of the genome and transcriptome assemblies, with an *E* value cutoff of 10^−1^. Best-scoring hits were compared to the NCBI(nr) protein database in order to filter out host and bacterial proteins. Additional mitochondrial contigs were then identified via BLASTn, using typical repetitive regions from contigs identified in the first step. *Perkinsela* nucleus-encoded proteins associated with mitochondrial oxidative phosphorylation, RNA editing and processing machineries, and mitochondrial translation were identified using HMMs (with an *E* value cutoff of 10^−10^), based on the corresponding orthologous groups from the OrthoMCL database realigned using MUSCLE (76). We classified as “missing” all *Perkinsela* hits with an *E* value of >10^−50^ that did not recover the corresponding *T. brucei* ortholog as the best hit in reciprocal BLASTp analysis (with an *E* value cutoff of 10^−3^). *Perkinsela* hits with an *E* value of <10^−50^ and without a suitable reciprocal BLASTp hit were aligned with their supposed orthologs in trypanosomatids. All protein alignments were performed using MUSCLE with default settings and checked manually.

### Searches for rRNAs.

The following approaches were used to identify mitochondrial rRNA genes in *Perkinsela*. First, BLAST searches with known kinetoplastid and diplonemid homologs as queries were performed with an *E* value cutoff of 10^−5^. Second, transcribed regions on contigs not assigned to the host or *Perkinsela* nuclear genomes were selected for further inspection. Third, reads containing the LSU peptidyl transferase core sequence (ACCTCGNTGT) conserved in *Diplonema* (16) were assembled separately using the CLC Genomics Workbench v.6.5. The top candidates from each of these searches were subjected to manual secondary structure folding, with terminal hairpin prediction performed using the Mfold thermodynamic folding application (http://mfold.rit.albany.edu/?q=mfold/RNA-Folding-Form). Default options were used to construct guiding graphs for manual secondary structure prediction (except for the “Loop max” option, which was restricted to 10, 20, and 30 nucleotides). Structures were assessed for similarity to those of *Leishmania* LSU and SSU and *Diplonema* LSU rRNAs (16, 22).

### Search for gRNA genes.

We implemented a simple search for putative gRNA genes in the following way: we reversed and complemented a final edited sequence of a transcript, cut it into 25-nt seeds with a seed step of 1 nt (taking only transcript regions undergoing RNA editing), searched for exact seed matches in the genome assembly and in its reverse complement, allowing up to 5 A-to-G or C-to-T substitutions in the 25-nt seed, in order to accommodate G-U base pairing. Then, we searched for RNA-seq reads that exactly matched the genomic hits, so that only transcribed hit regions were taken.

### Bowtie2 modification.

Bowtie2 is an open-source, fast, and accurate short read mapper written in the C++ programming language (77). It uses a fast multiseeding procedure to find candidate alignment locations and then proceeds with the Smith-Waterman algorithm to create the best gapped alignment. For additional speed, Bowtie2 implements the Smith-Waterman alignment algorithm with SIMD (single instruction, multiple data), allowing it to fill several dynamic programming table cells by executing a single instruction (78). However, Bowtie2 uses a scoring system with equal gap open and extension penalties for the four nucleotides, A, G, T, and C. We modified Bowtie2 to facilitate accurate alignment of U insertion/deletion edited RNA reads, while preserving mapping speed and accuracy. Edited reads of the mitochondrial genomes of kinetoplastids have U-indels only, and therefore they can be aligned correctly when gap penalties for Ts (corresponding to Us in RNA) are different from those for A, G, and C.

We modified the Bowtie2 v.2.0.2 source code and implemented a more complex nucleotide-specific gap-scoring system that allowed separate penalty values for A, G, T, and C by using the −rdg-X and −rfg-X options on the command line (for gaps in the read and reference, respectively, where X can be A, T, G, or C). Source code modifications were made both in the aligner module, which fills the dynamic programming table, and in the backtrack module of the program, which reconstructs the alignment using the filled dynamic programming table. Branch and array access instructions were minimized for each step, ensuring minimal time cost for more complex scoring. Using this scoring matrix, U-indel edited reads can be successfully mapped and accurately aligned with a low T-indel penalty and high penalties for other nucleotides. Additional modifications of the alignment procedure were necessary in order to let reads have a gap/mismatch after the last nucleotide of the read (option gbar 0). This option allows the seeding of more extensively edited reads on a preedited RNA sequence and prevents a significant fraction of edited reads from being discarded.

### T-aligner.

T-aligner is a new software program written for the purpose of this study and we used the C++ programming language (source code posted online at https://github.com/jalgard/T-Aligner). T-aligner combines the optimal but time-consuming Smith-Waterman alignment with fast hash-based exact matching. The algorithm is specially designed to map extensively edited RNA-seq reads on pre-edited transcript references, also called cryptogenes. Exact matches between short substrings (seeds) are first found using a hash table. A local optimal alignment is then produced with the Smith-Waterman algorithm, allowing “T, −” and “−, T” gaps with zero penalty, thus taking into account the biological mechanism of U-indel RNA editing. The general T-aligner workflow is as follows (see also Fig. S4 in the supplemental material): a fixed seed is chosen in a never-edited or universally edited 3′-terminus of the transcript (or editing domain, in appropriate cases). Reads are then mapped if they satisfy the following criteria: (i) they contain the seed; (ii) at least part of the read lies 5′ to the seed; (iii) the alignment may contain any number of U-indels of any length; (iv) the alignment contains no other indels and no or few mismatches. After the alignments are produced, T-aligner classifies all editing events (U insertion or U deletion) and clusters the reads into three groups: (i) those matching the reference sequence, (ii) those matching the putative main “editing pathway” (i.e., the user-defined final edited product), and (iii) all other reads containing alternative editing events. Reads matching the main pathway are defined as follows: (i) those with no additional edited sites compared to the main pathway; (ii) reads with insertions/deletions that are shorter or equal in size to those in the main pathway; and (iii) reads in which all sites are edited in the same direction as in the main pathway (e.g., insertion in the main pathway versus insertion in a sequence read). Reads in violation of any of these conditions are placed in the “alternative editing” group. Sequence reads that are exact substrings of other reads are then merged into “editing intermediates.” The support value associated with an editing intermediate can be used to determine the most abundant sequences, which is useful when examining alternative editing. All sequences clustered into the “reference” and “main pathway” groups are assigned a support value equal to the number of reads in each group. For each sequence from the “alternative” group, support is determined as follows: reads falling into the “reference” and “main pathway” groups are excluded; if a read is unique, i.e., can be included as a substring in at most one longer read, it adds 1 to the support value; if a read supports *k* > 1 alternative sequences, it adds 1/*k* to a support value for each sequence.

### Read mapping and analysis of U-indel RNA editing.

Bowtie2 v.2.0.2 or v.2.1.0 mapping software was used for both DNA and RNA-seq reads, and we utilized the end-to-end mapping mode, the “very sensitive” options, and default alignment scoring. In order to produce precise alignments in extensively edited regions, we used a modification of Bowtie2 v.2.0.2 with the base-specific indel penalties described above. The following set of options was routinely used: (i) high gap opening and extension penalties of 10 for A, G, or C in the reference and individual sequence reads (--rfg 10,10; --rdg 10,10); (ii) minimal gap opening and extension penalties of 1 for T or A (depending on transcript orientation) in the reference and reads (--rfg-T 1,1; --rdg-T 1,1 or --rfg-A 1,1; --rdg-A 1,1); (iii) a high mismatch penalty equal to 18 (--mp 18); (iv) options allowing terminal mismatches (gbar 0; dpad 50), and (v) other options (--end-to-end; -D 20; -R 3; -N 1; -L 14; -i S,1,0.50; --score-min L,0,-2). Reads mapped to the edited regions were manually checked before further processing. Poor-quality alignments, especially those introducing large gaps, were not considered. Alignments made with Bowtie2 were cut into overlapping windows and examined to find sequences appropriate for seeding further read mappings with T-aligner.

One to three iterations of read mapping with T-aligner (with the original seed shifting in the 3′-to-5′ direction) were enough to cover the whole transcript or its edited region and then reconstruct the main editing pathway. Repeating T-aligner-assisted read mapping with prior knowledge of the main edited product allowed us to reveal and quantify alternative editing products.

### Northern blotting.

Northern blotting analysis of *cox2* was performed as previously described (59). Briefly, 10 µg of RNA isolated from *Perkinsela* strain GillNOR1/I and *T. brucei* strain 29-13 was run on a high-resolution 4% acrylamide–7 M urea gel and transferred onto a Zeta-probe membrane (Bio-Rad). The membrane was subsequently probed with 5′-^32^P-labeled oligonucleotides corresponding to the antisense (5′-CCCTTTCAACACGTCAAAACAAGC-3′) and sense (5′-GCTTGTTTTGACGTGTTGAAAGGGC-3′) pre-edited sequences of the 5′ end, i.e., the last to be processed, of the larger 3′-edited domain. The oligonucleotides were also used to probe dot blots of serially diluted, denatured PCR products amplified from this same region, in order to demonstrate that the two probes were equally sensitive.

## SUPPLEMENTAL MATERIAL

Figure S1  *Perkinsela* strain GillNOR1/I mitochondrial scaffolds with both sense and anti-sense transcriptomic reads mapped. Almost no antisense transcription is visible, which is supported by the Northern blot in Fig. 3. The sense transcription profile, showing very low coverage for pan-edited genes *rps12* and *atp6*, is different from that shown in Fig. 2 since “U-indel optimized” settings in Bowtie were not used here. “U-indel optimized” settings may produce strand biases, e.g., favoring U-indels on the forward strand, but not A-indels on the reverse strand. Therefore they were not used for the purpose of inter-strand comparison of transcription profiles. However, regular Bowtie “very sensitive” settings produce especially poor coverage in the case of pan-edited transcripts. Download Figure S1, TIF file, 4.9 MB

Figure S2  Mapping of edited reads with Bowtie2 v.2.0.2 and its modified version. An alignment window shown here covers the 3′ editing region of *cox1* in *Perkinsela* strain GillNOR1/I. Just a few reads are mapped by the standard Bowtie2 algorithm using the “very-sensitive” setting. In contrast, modified Bowtie2 with T-indel-sensitive settings results in 12-fold increase of mapped read count (not all reads are shown in the figure). Moreover, misalignments such as those shown with arrows are missing because gaps containing ACG are penalized. Download Figure S2, TIF file, 2.98 MB

Figure S3  All reads spanning both 5′ and 3′ edited domains of *cox2* in *Perkinsela* strain GillNOR1/I. Only parts of the edited domains adjoining the central non-edited region are shown, and the non-edited region itself is omitted (represented by the black bar in the center of the picture). The pre-edited sequence is shown at the bottom and the final edited sequence is on top. Insertions are shown in light blue, deletions in red, and edits corresponding to the main edited product are boxed (alternative edits are not boxed). We found virtually no reads edited in the 3′ domain but not edited in the 5′ domain, but many examples of the opposite arrangement. Only a single read carries one alternative edit in the 3′ domain and no other edits. Download Figure S3, TIF file, 7.36 MB

Figure S4  Workflow of T-aligner. Download Figure S4, TIF file, 1 MB

File S1 Alternatively edited and “misedited” products for *cox1*, *cox2*, *cox3*, *cob*, *atp6*, and *rps12*. The figure represents an output of T-aligner, illustrating non-redundant alternatively edited reads (also termed "editing intermediates" in this paper to distinguish them from reads in the raw dataset). Each column represents an editing site irrespective of the U-indel length observed; each line represents a read with its sequence in white. U-insertion and U-deletion sites identical to those in the main edited product are shown in dark green and dark red, respectively. A site is considered alternatively edited if any of the following conditions are met: (i) a site is never-edited in the main editing product; (ii) U indel is longer than in the main product; (iii) U insertion occurs instead of deletion in the main product or vice versa. Alternatively edited sites are shown in bright green and orange for U insertions and U deletions, respectively, and sites with the editing direction reversed (group iii) are denoted in blue. The transcript is schematically shown at the bottom, with edited domains in green and the seed used by T-aligner in yellow. Download File S1, DOCX file, 0.1 MB

File S2 Genomic and transcriptomic NGS reads. Paired transcriptomic reads were merged with the CLC Genomics Workbench v.6.5 prior to mapping. Merged read length distribution is shown for the *Perkinsela* CCAP1560/4 (A) and GillNOR1/I strains (B). The abrupt edges of the distribution in panel A are due to 100 bp and shorter trimmed reads which produce merged reads of 190 bp or shorter, if a minimum overlap of 10 bp is required. Download File S2, DOCX file, 3.39 MB

File S3 Guide RNA gene candidates revealed in the genome assembly of *Perkinsela* sp. CCAP1560/4. Aligned stretches of gRNA and mRNA (at least 25 bp in length) are shown in uppercase, and their flanks are shown in lowercase. Contig IDs and start coordinates are shown, along with a number of matching transcriptomic reads (support). The 5′ AC-rich region of gRNAs is underlined. Download File S3, DOCX file, 0.5 MB

File S4 Aligned edited/pre-edited transcripts and trees for the final protein sequences of 6 mitochondrial genes in *Perkinsela* strains CCAP1560/4 and GillNOR1/I. For each gene (*cox1*, *cox2*, *cox3*, *cob*, *rps12*, and *atp6*) the following information is shown: (i) edited and pre-edited transcript sequences with corresponding translations (U-insertions are denoted in blue and U-deletions are marked with orange circles); (ii) pairwise percent identities (in the lower left part of the matrix) and numbers of different positions (in the upper right part) between edited/pre-edited sequences of both strains; (iii) a maximum likelihood unrooted tree for protein sequences of *Perkinsela*, other kinetoplastids, and an outgroup (*Diplonema papillatum*, *Euglena gracilis*, or *Naegleria gruberi*, depending on sequence availability). The trees were constructed using the following settings: WAG+Г substitution model, neighbor-joining starting tree, 1,000 bootstrap replicates. Branches supported by bootstrap values >70% are shown with thicker lines. Scale bars show inferred number of amino acid substitutions per site. Download File S4, DOCX file, 3.47 MB

File S5 Alternative editing quantification. Pie charts illustrating counts of reads matching the main editing pathway, the pre-edited sequence, and alternatively edited reads in *Perkinsela* strains CCAP1560/4 and GillNOR1/I (A). Alternative editing intermediates in *Perkinsela* sp. Mitochondria, sorted according to their rounded support values (B) and number of editing sites (C) (x-axis) are shown. Read counts in logarithmic scale (y-axis) are given separately for each model edited domain (strain CCAP1560/4 for *cox1*, *cox3*, *atp6*; strain GillNOR1/I for *cox2*, *cob*, *rps12*). Download File S5, DOCX file, 0.1 MB

Table S1  *Perkinsela* nucleus-encoded proteins associated with mitochondrial oxidative phosphorylation, RNA editing and processing machineries, and mitochondrial translation, identified using HMM and reciprocal BLASTp. Table S1, XLSX file, 0.01 MB
