# Supplementary figures and images for: Gene Loss and Error-Prone RNA Editing in the Mitochondrion of Perkinsela, an Endosymbiotic Kinetoplastid
Source: mBio. 2015 Dec 1;6(6):e01498-15. doi: 10.1128/mBio.01498-15 (PMC4669381; doi:10.1128/mBio.01498-15)

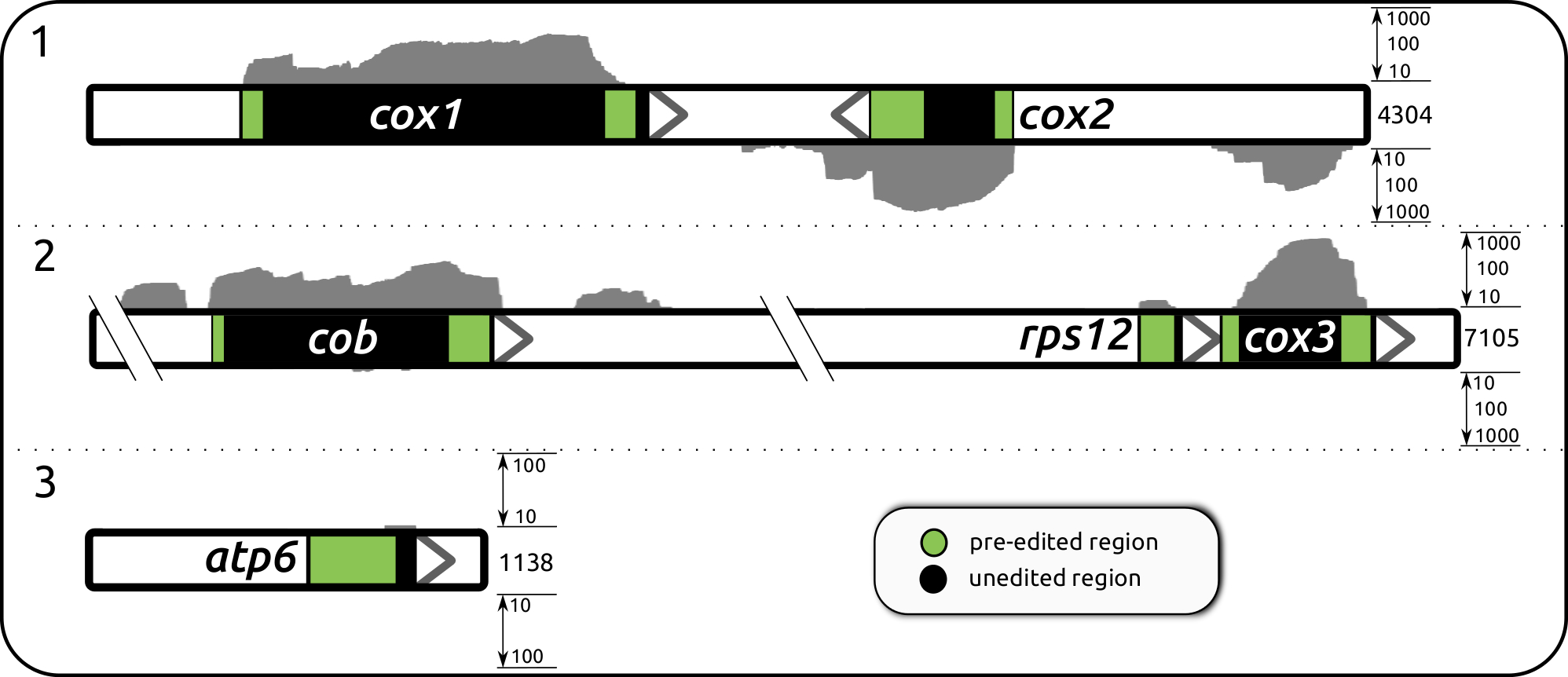

Supplement: Figure S1 — Perkinsela strain GillNOR1/I mitochondrial scaffolds with both sense and anti-sense transcriptomic reads mapped. Almost no antisense transcription is visible, which is supported by the Northern blot in Fig. 3. The sense transcription profile, showing very low coverage for pan-edited genes rps12 and atp6, is different from that shown in Fig. 2 since “U-indel optimized” settings in Bowtie were not used here. “U-indel optimized” settings may produce strand biases, e.g., favoring U-indels on the forward strand, but not A-indels on the reverse strand. Therefore they were not used for the purpose of inter-strand comparison of transcription profiles. However, regular Bowtie “very sensitive” settings produce especially poor coverage in the case of pan-edited transcripts. Download [file mbo005152537sf1.tif]

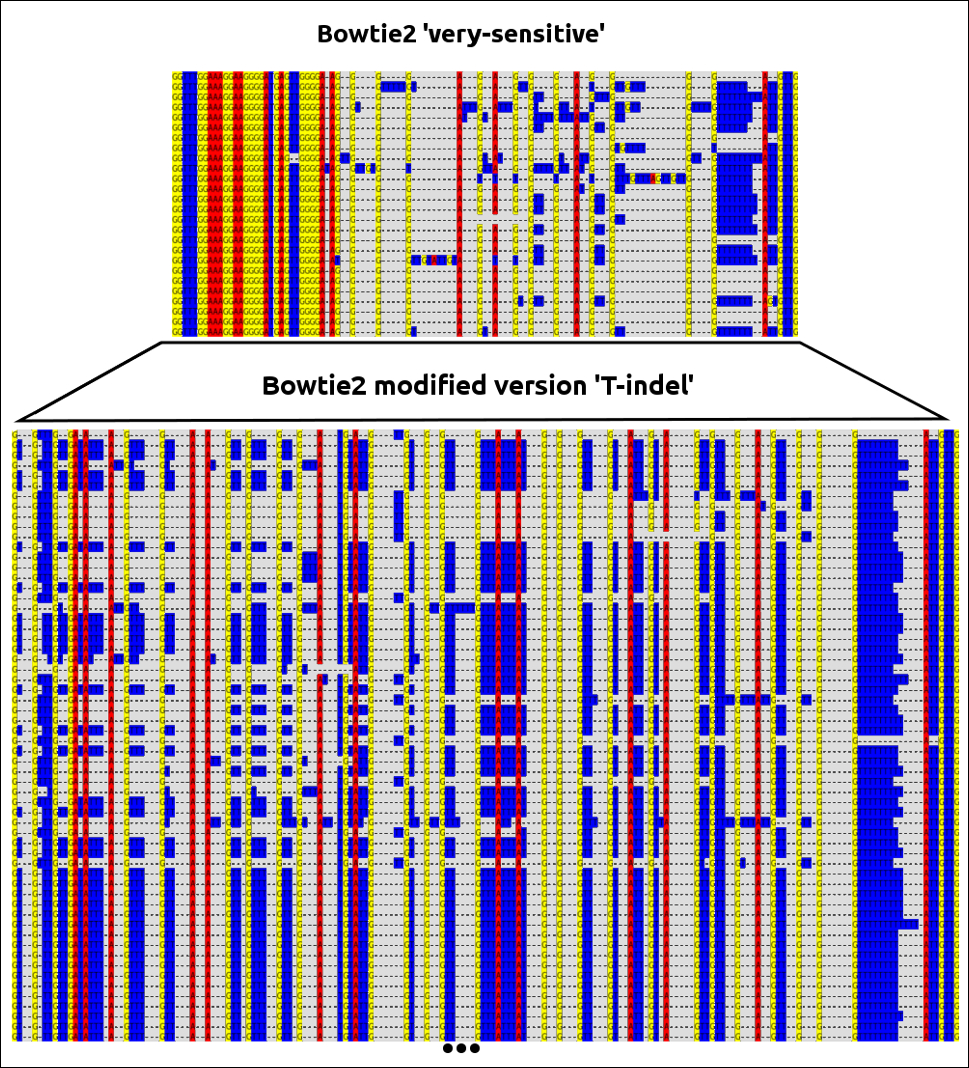

Supplement: Figure S2 — Mapping of edited reads with Bowtie2 v.2.0.2 and its modified version. An alignment window shown here covers the 3′ editing region of cox1 in Perkinsela strain GillNOR1/I. Just a few reads are mapped by the standard Bowtie2 algorithm using the “very-sensitive” setting. In contrast, modified Bowtie2 with T-indel-sensitive settings results in 12-fold increase of mapped read count (not all reads are shown in the figure). Moreover, misalignments such as those shown with arrows are missing because gaps containing ACG are penalized. Download [file mbo005152537sf2.tif]

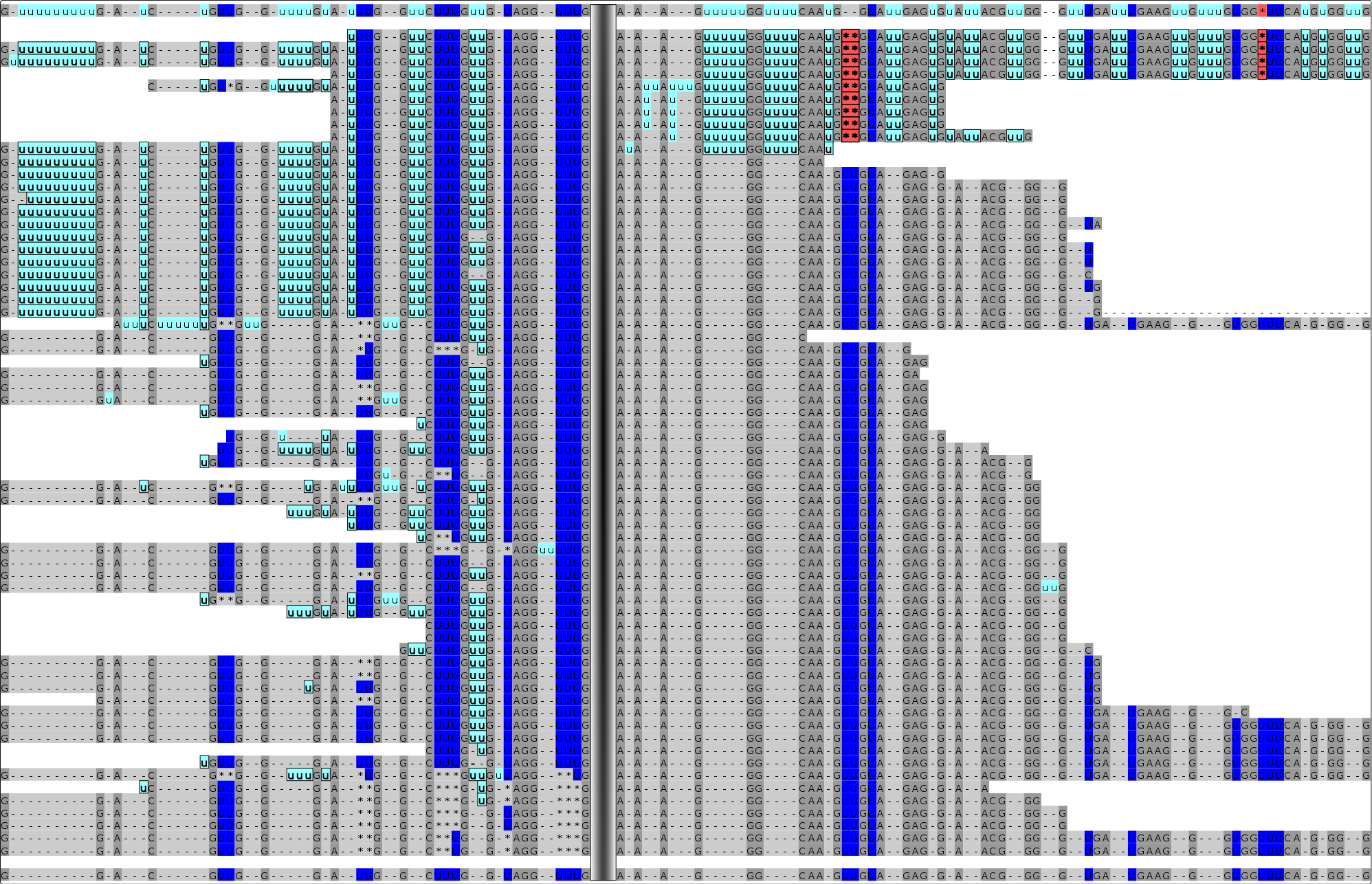

Supplement: Figure S3 — All reads spanning both 5′ and 3′ edited domains of cox2 in Perkinsela strain GillNOR1/I. Only parts of the edited domains adjoining the central non-edited region are shown, and the non-edited region itself is omitted (represented by the black bar in the center of the picture). The pre-edited sequence is shown at the bottom and the final edited sequence is on top. Insertions are shown in light blue, deletions in red, and edits corresponding to the main edited product are boxed (alternative edits are not boxed). We found virtually no reads edited in the 3′ domain but not edited in the 5′ domain, but many examples of the opposite arrangement. Only a single read carries one alternative edit in the 3′ domain and no other edits. Download [file mbo005152537sf3.tif]

***cox1***


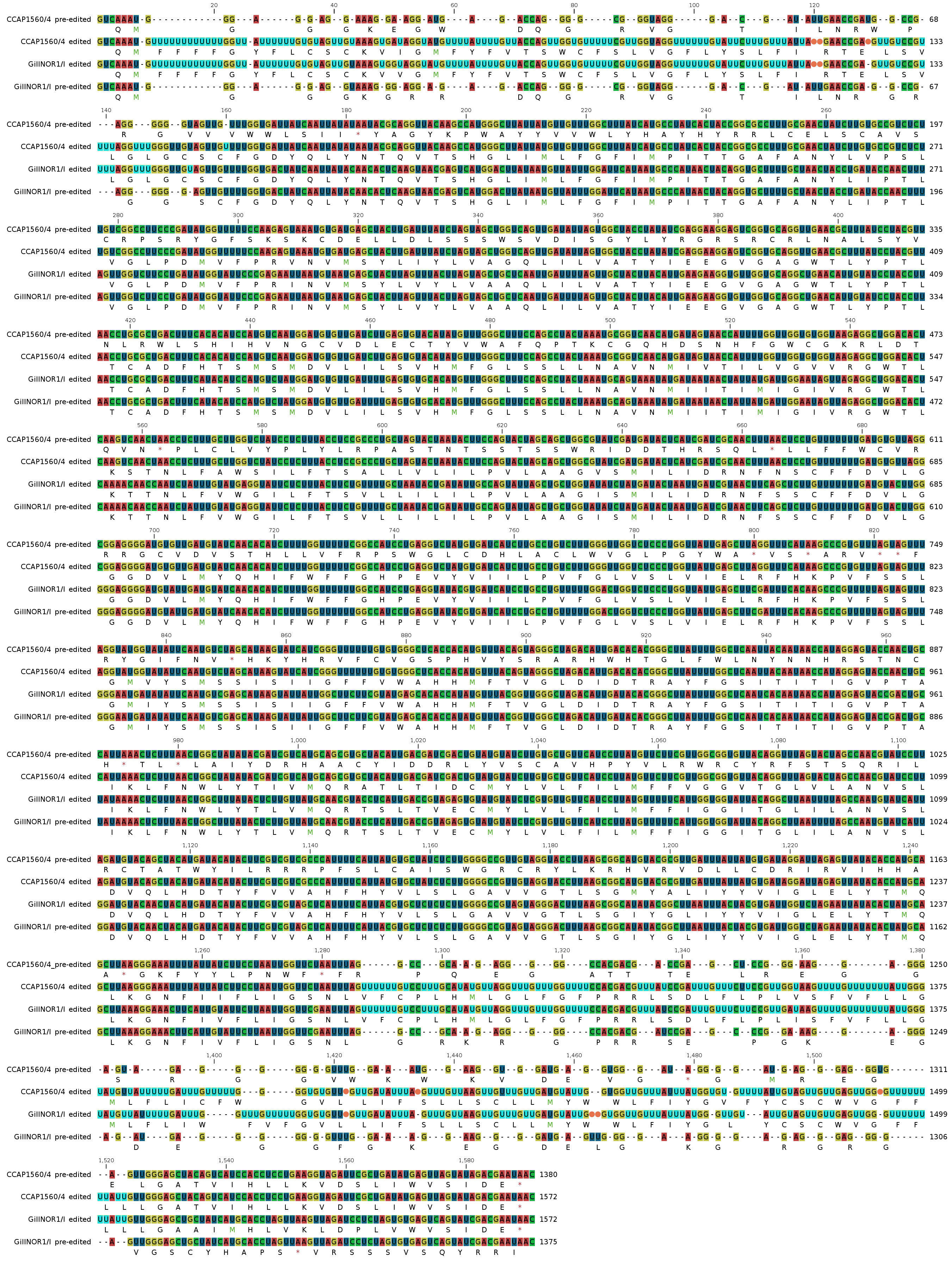


**
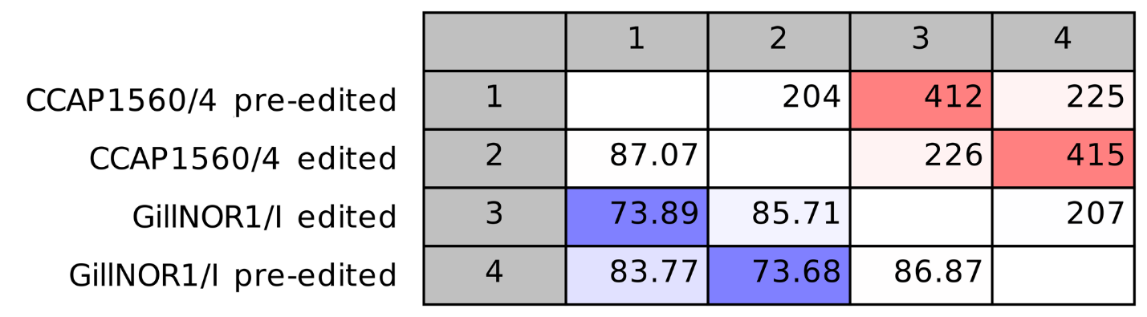
**

**
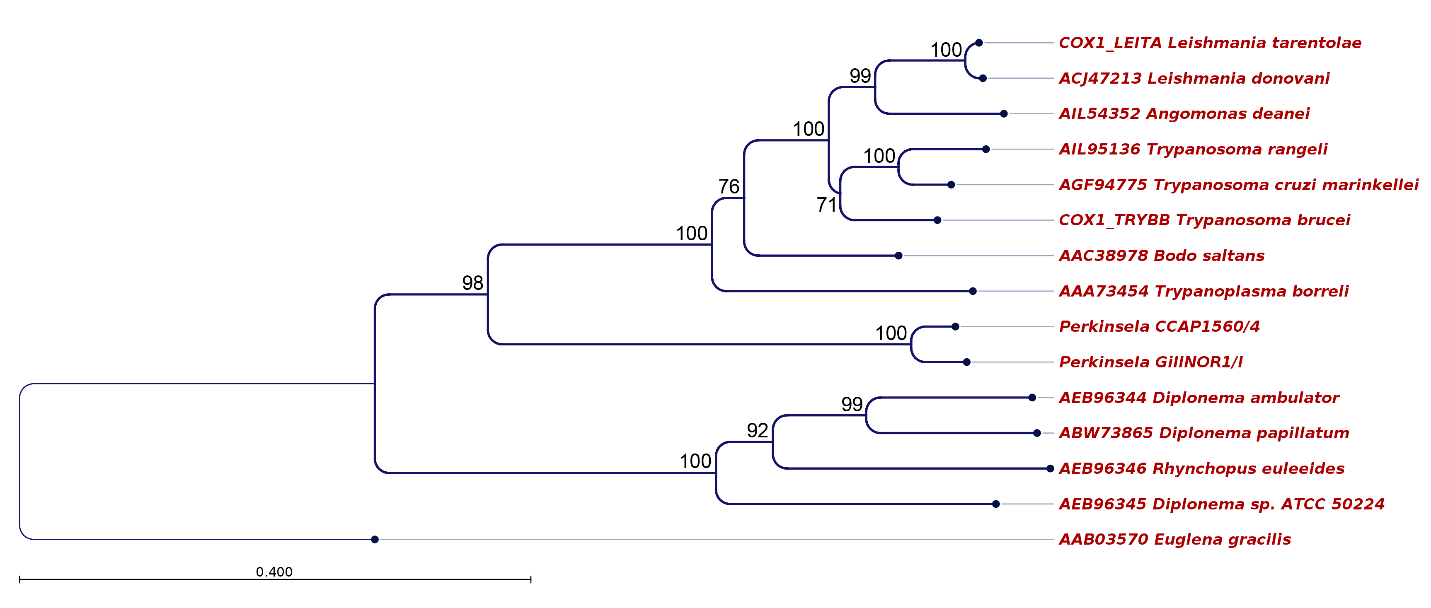
**

***cox2***

**
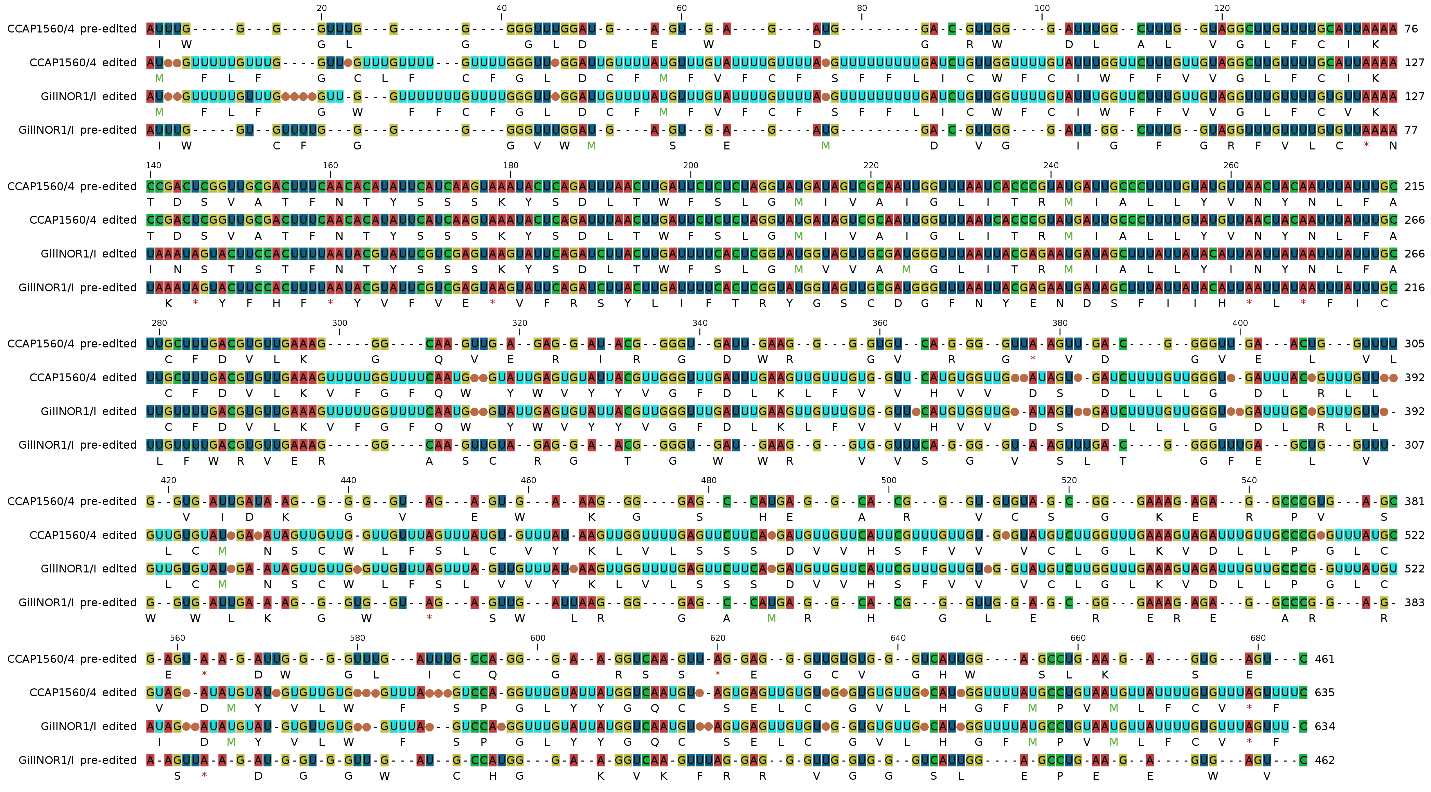
**

**
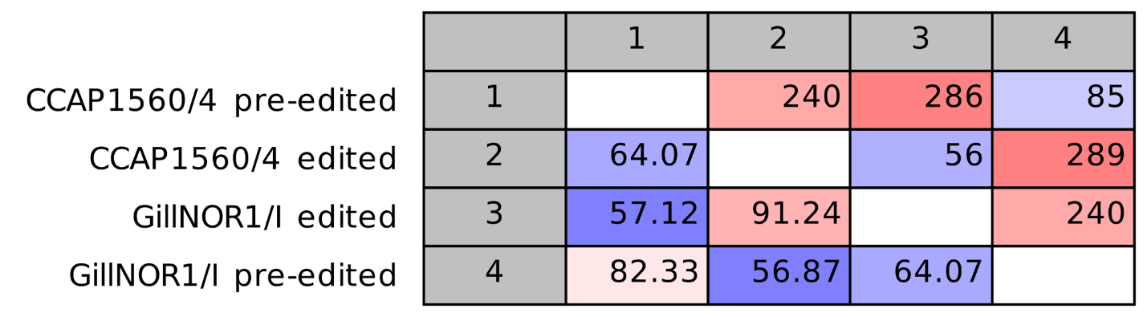
**

**
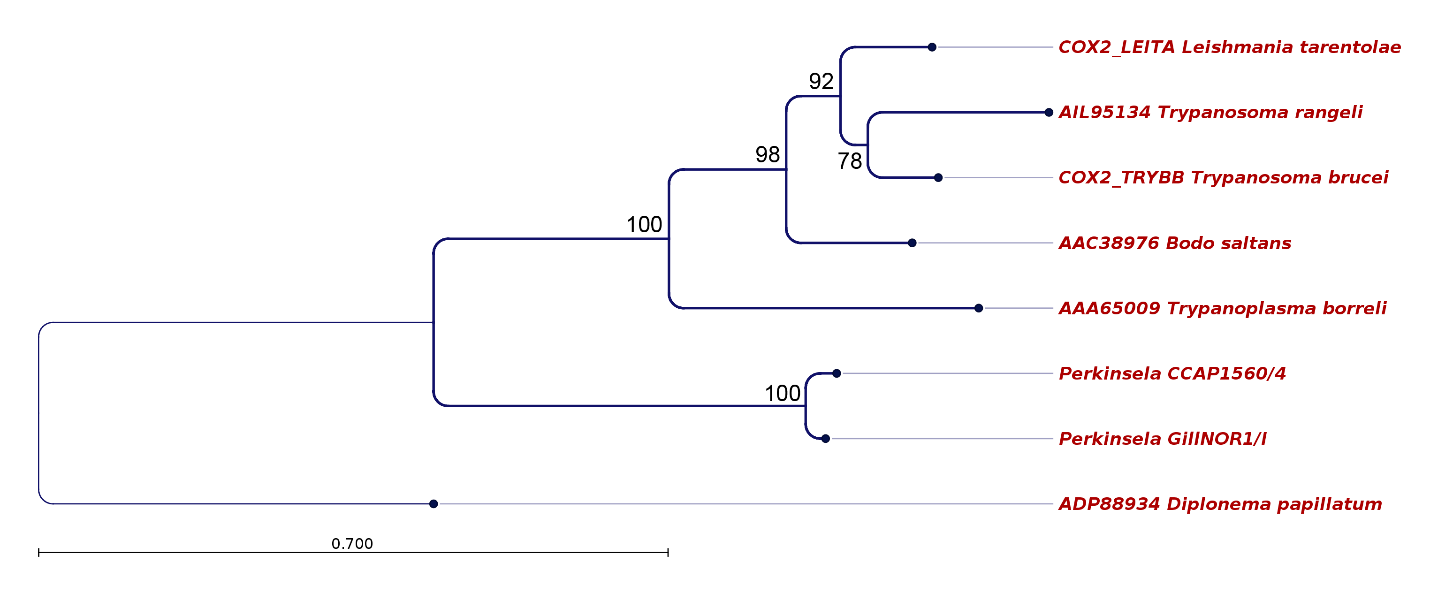
**

***cox3***

**
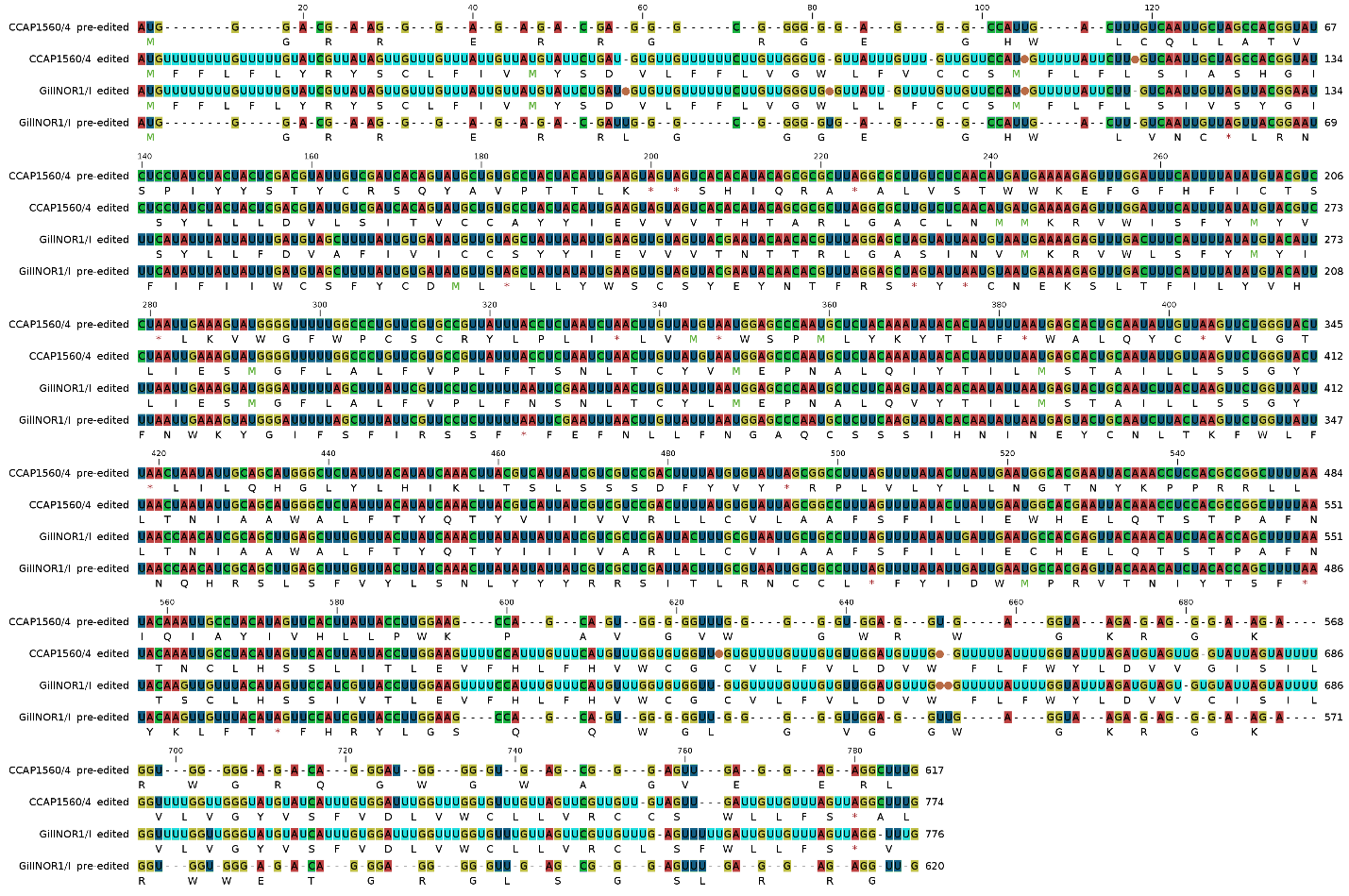
**

**
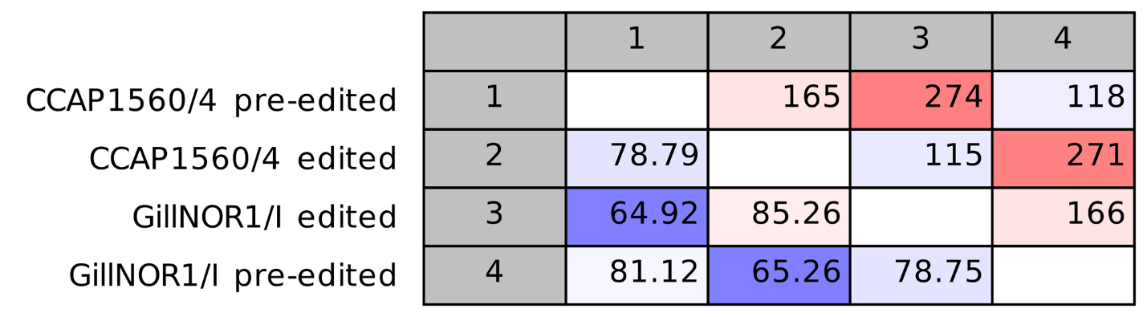
**

**
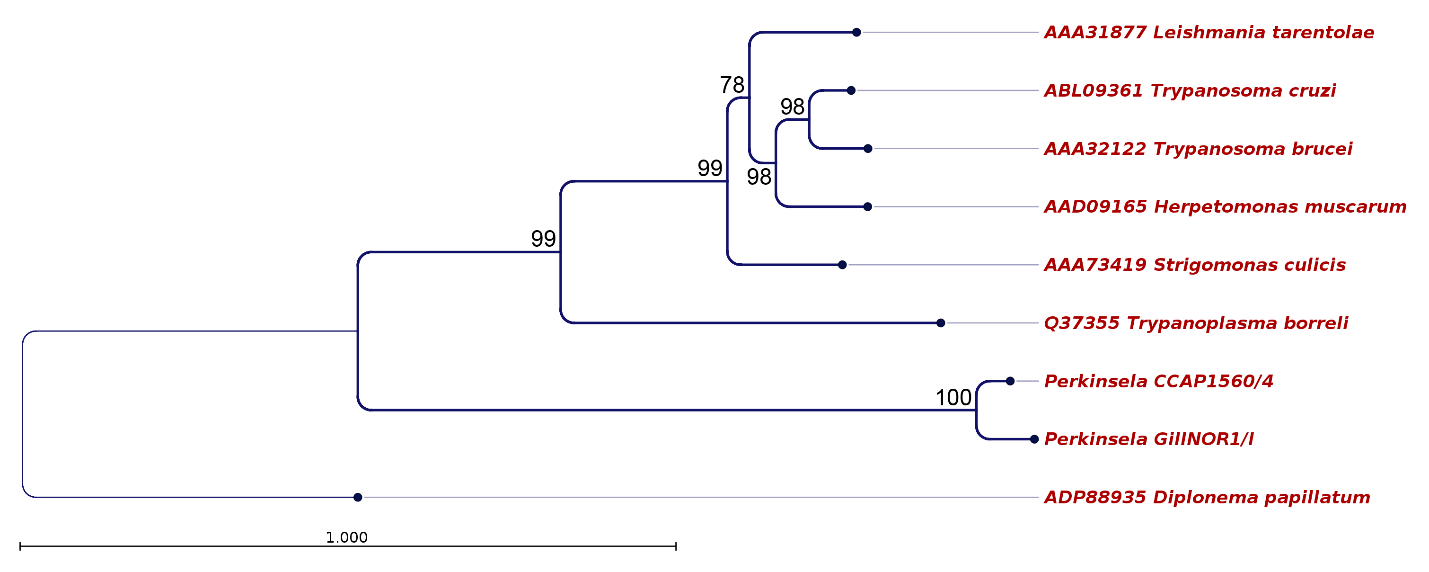
**

***cob***

**
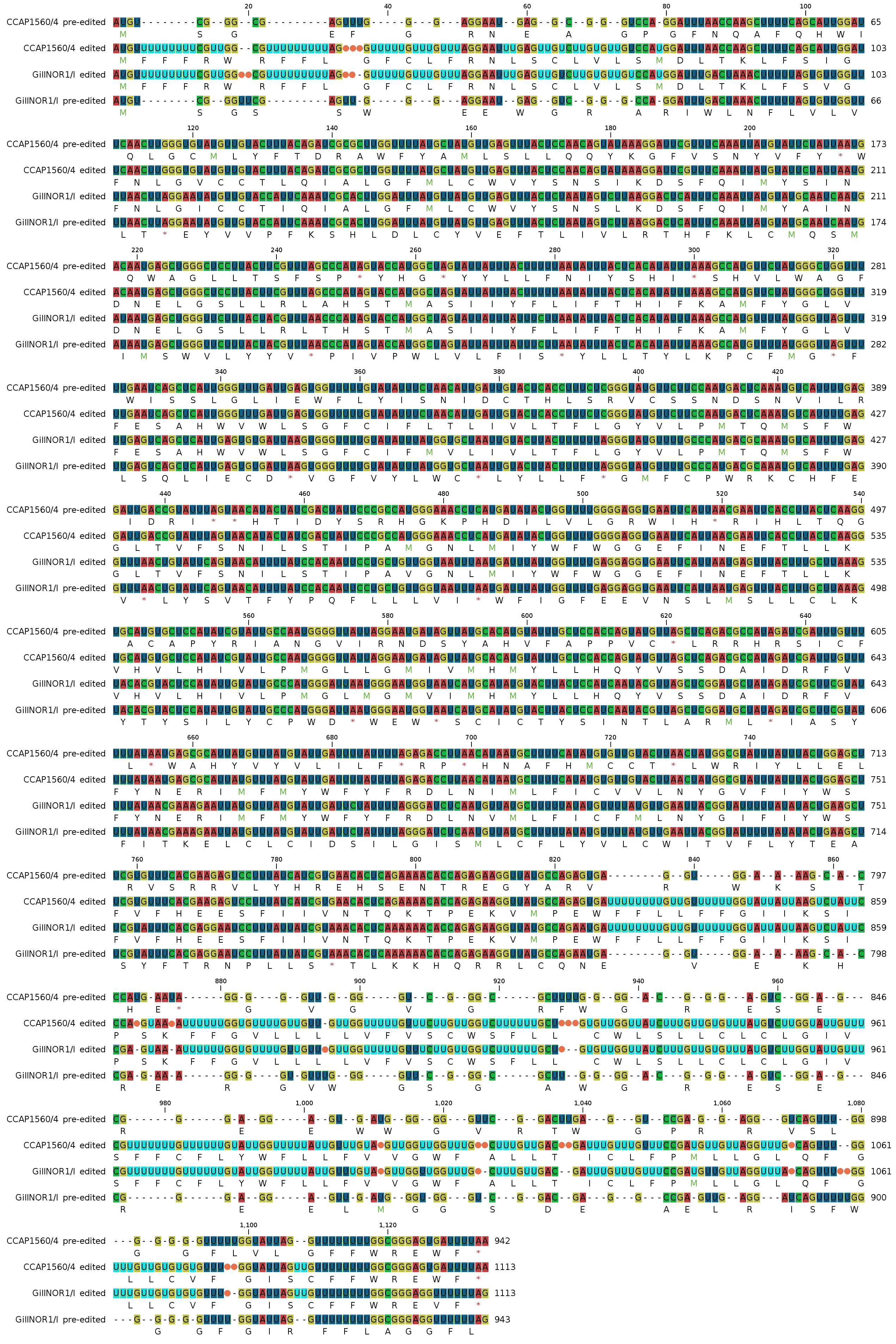
**

**
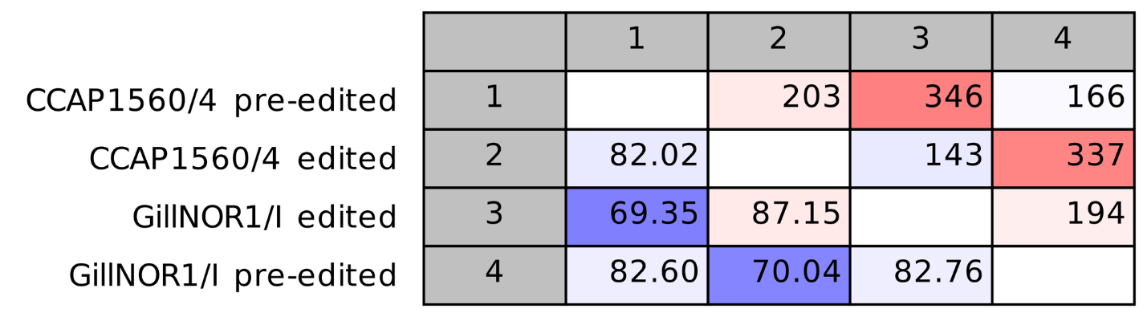
**

**
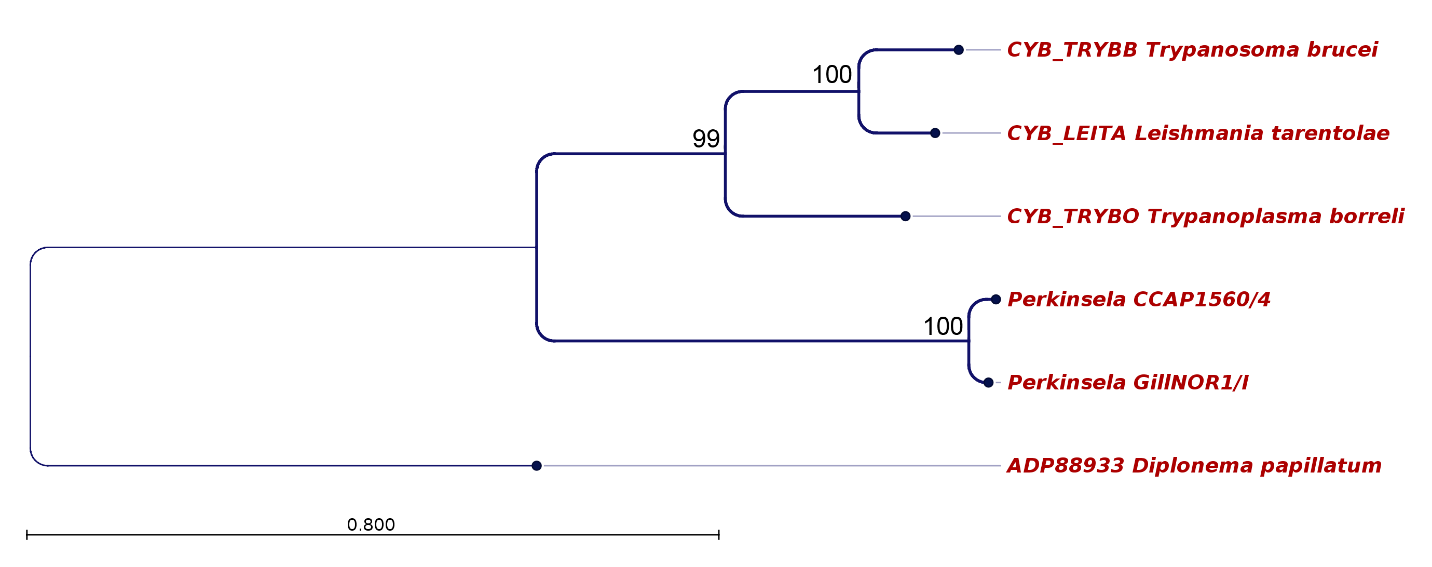
**

***rps12***

**
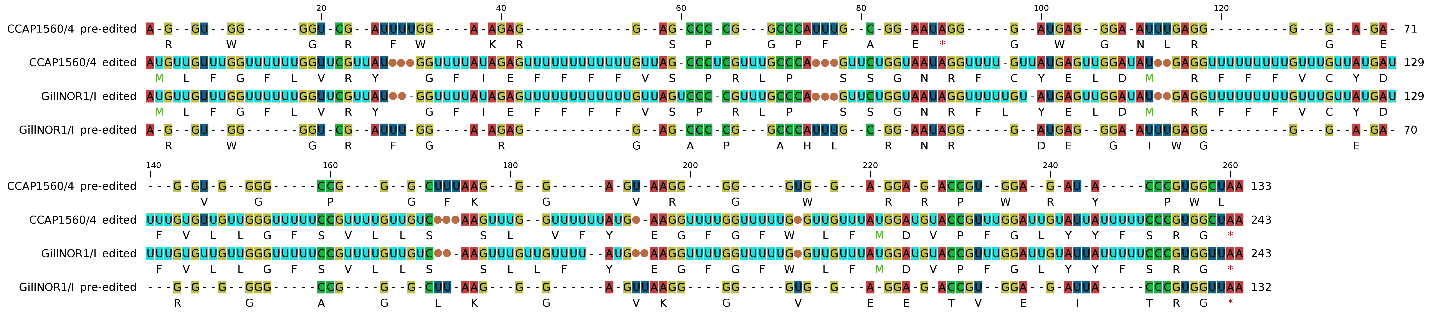
**

**
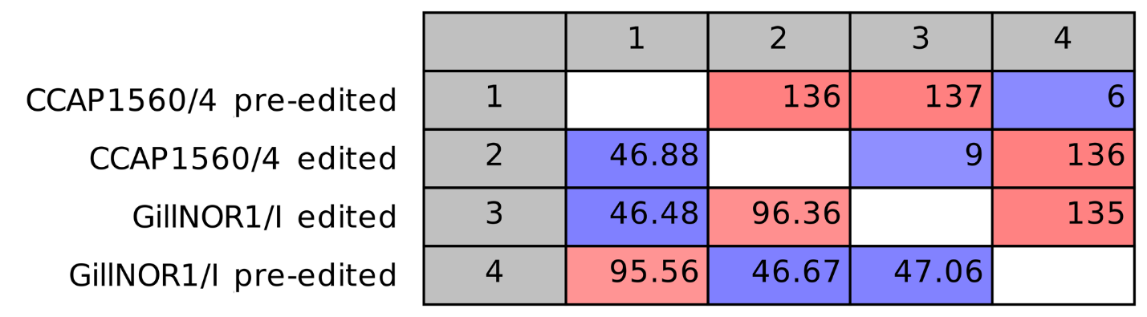
**

**
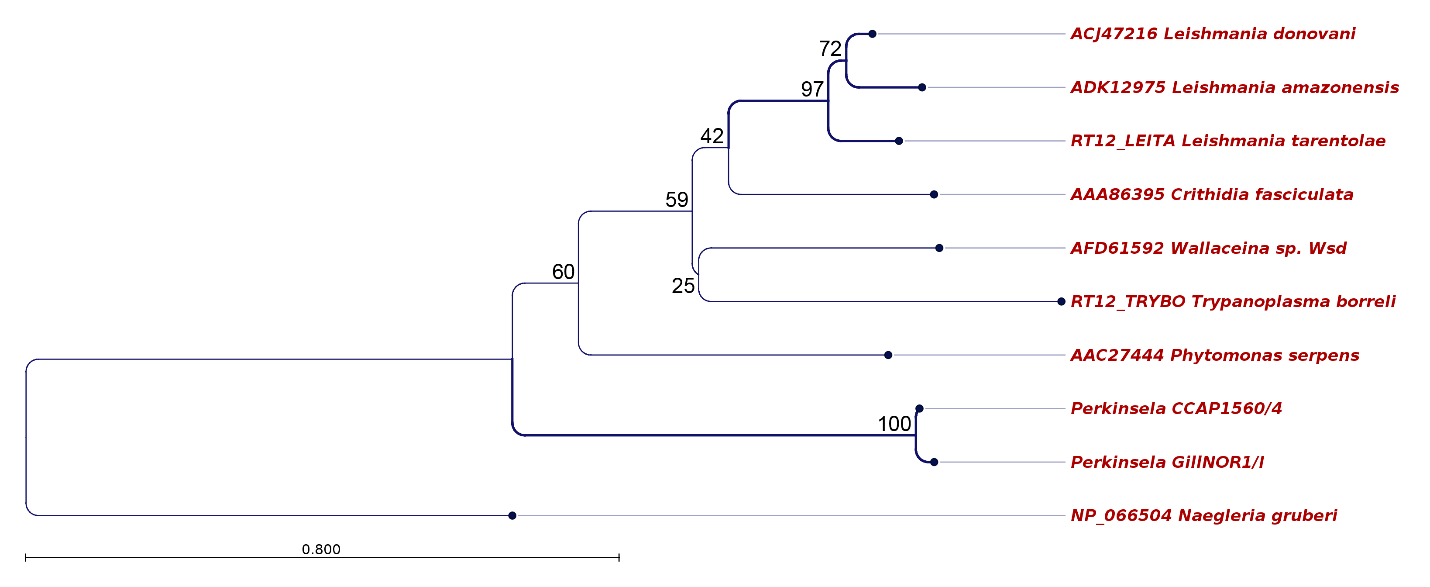
**

***atp6***

**
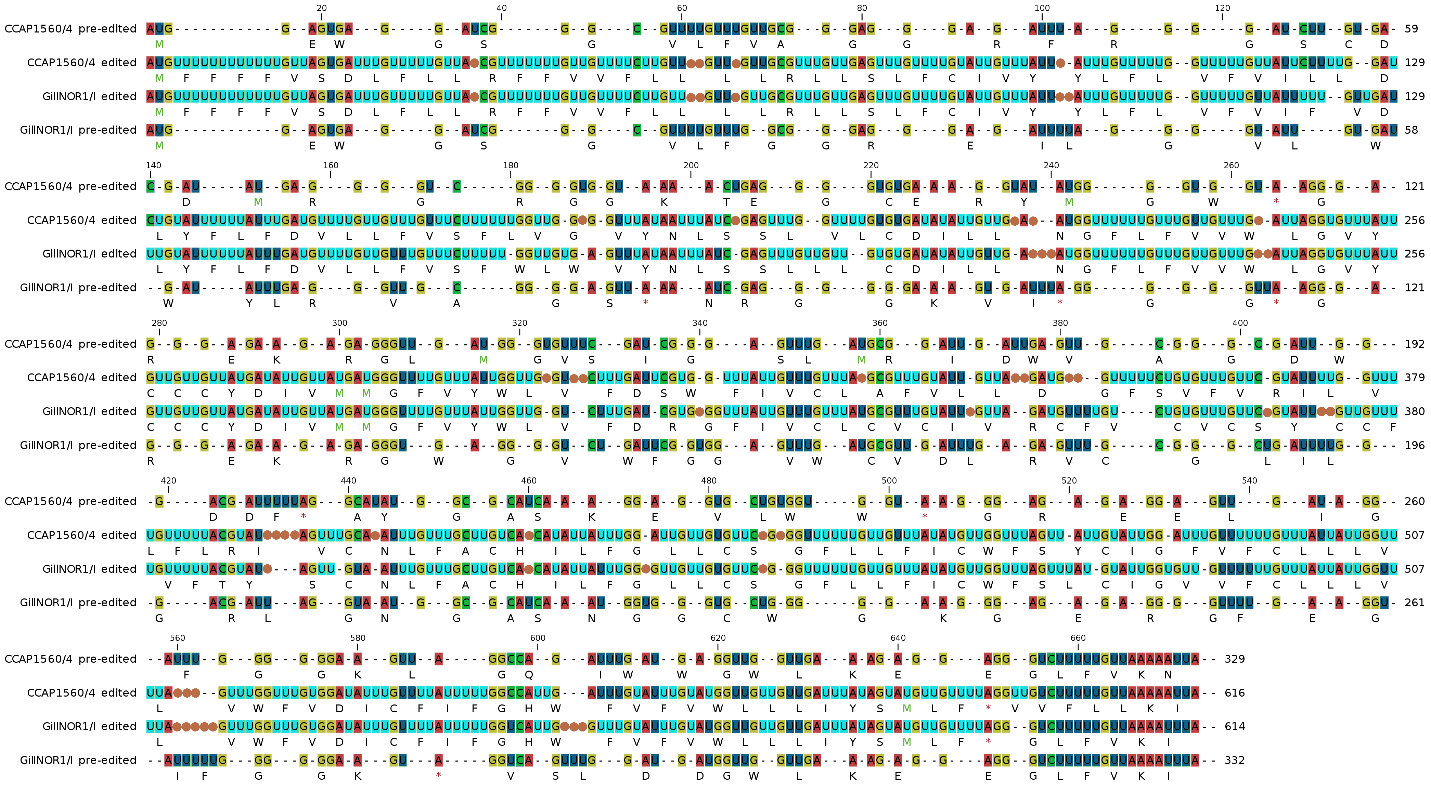
**

**
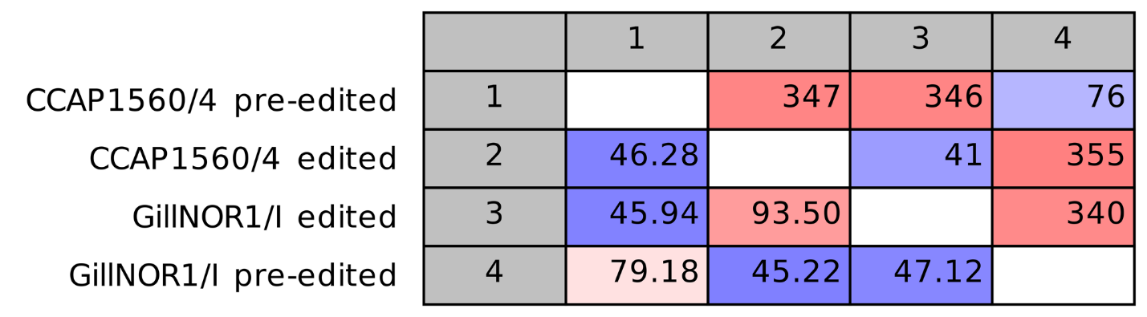
**

**
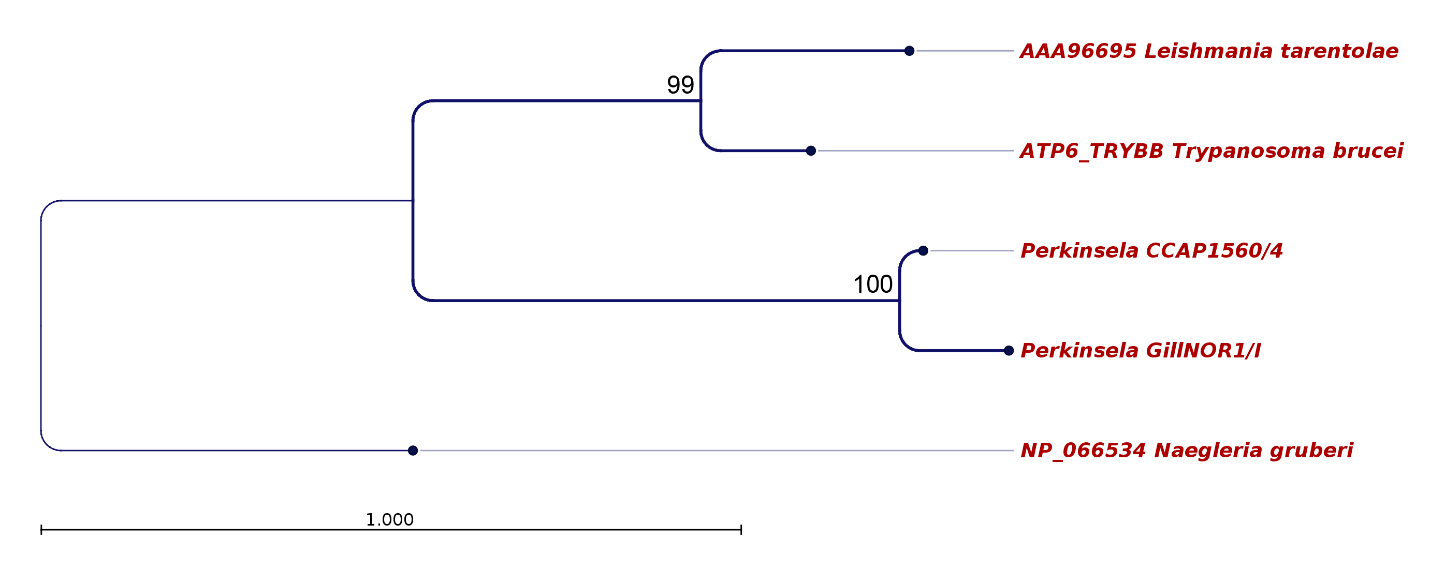
**

Supplement: File S4 — Aligned edited/pre-edited transcripts and trees for the final protein sequences of 6 mitochondrial genes in Perkinsela strains CCAP1560/4 and GillNOR1/I. For each gene (cox1, cox2, cox3, cob, rps12, and atp6) the following information is shown: (i) edited and pre-edited transcript sequences with corresponding translations (U-insertions are denoted in blue and U-deletions are marked with orange circles); (ii) pairwise percent identities (in the lower left part of the matrix) and numbers of different positions (in the upper right part) between edited/pre-edited sequences of both strains; (iii) a maximum likelihood unrooted tree for protein sequences of Perkinsela, other kinetoplastids, and an outgroup (Diplonema papillatum, Euglena gracilis, or Naegleria gruberi, depending on sequence availability). The trees were constructed using the following settings: WAG+Г substitution model, neighbor-joining starting tree, 1,000 bootstrap replicates. Branches supported by bootstrap values >70% are shown with thicker lines. Scale bars show inferred number of amino acid substitutions per site. Download [file mbo005152537s4.docx]

**
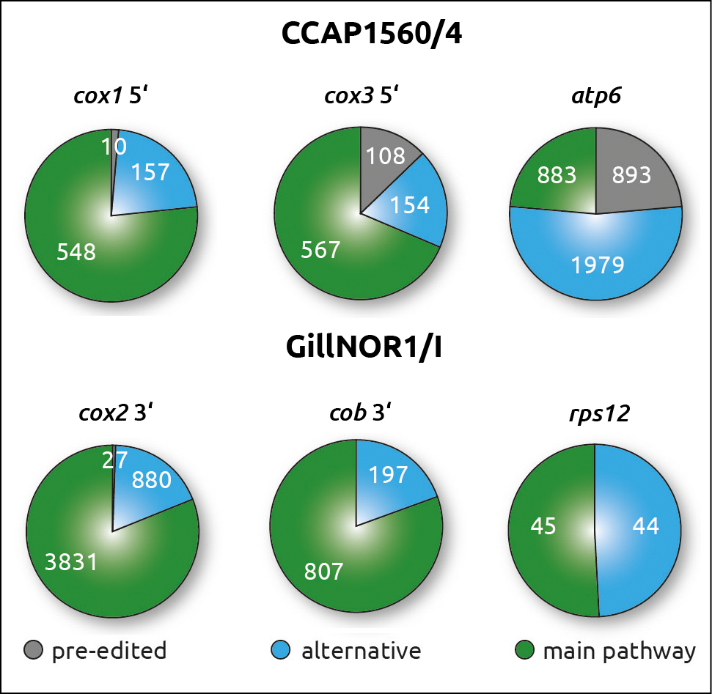
A**

**BC**


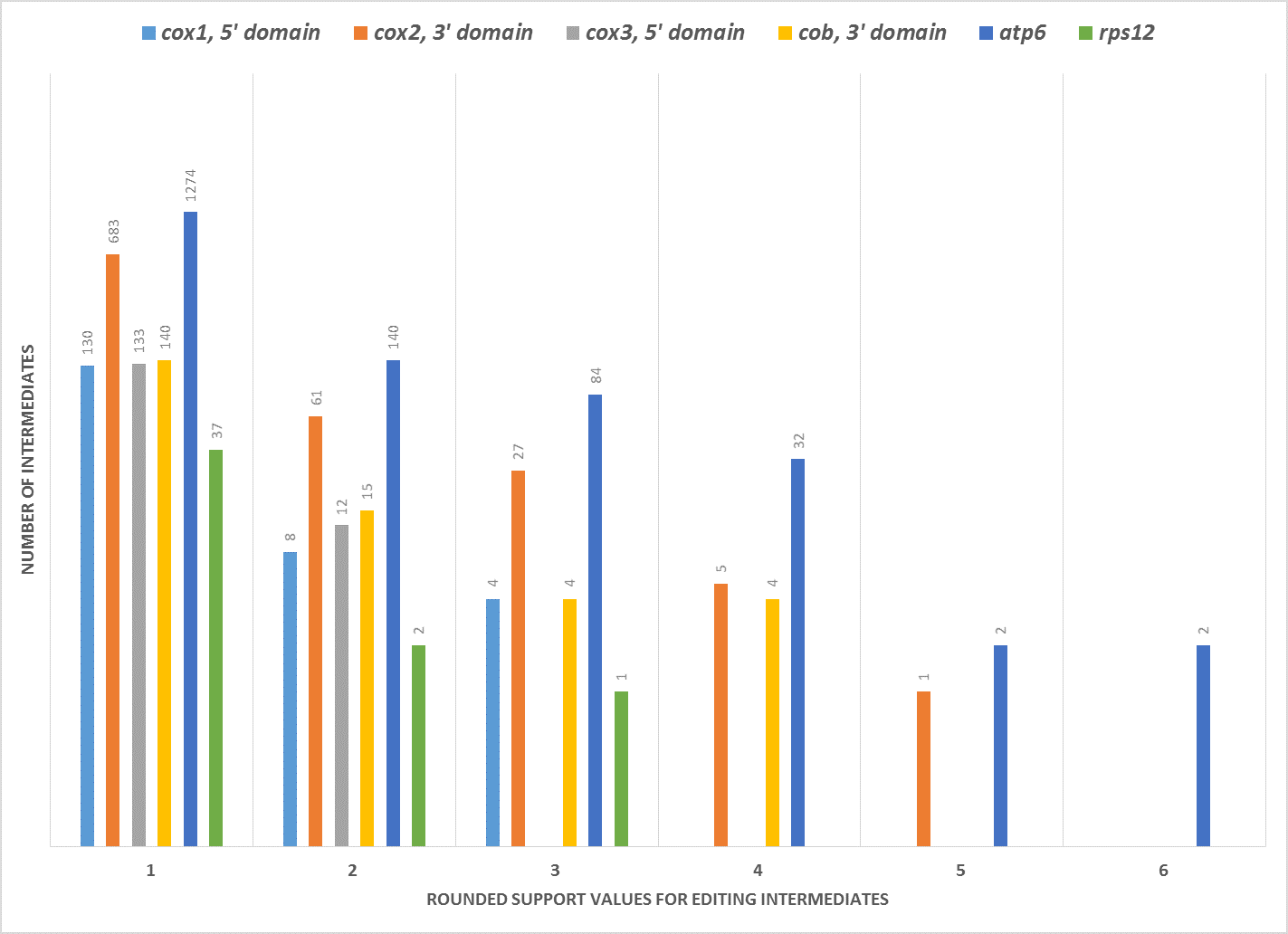

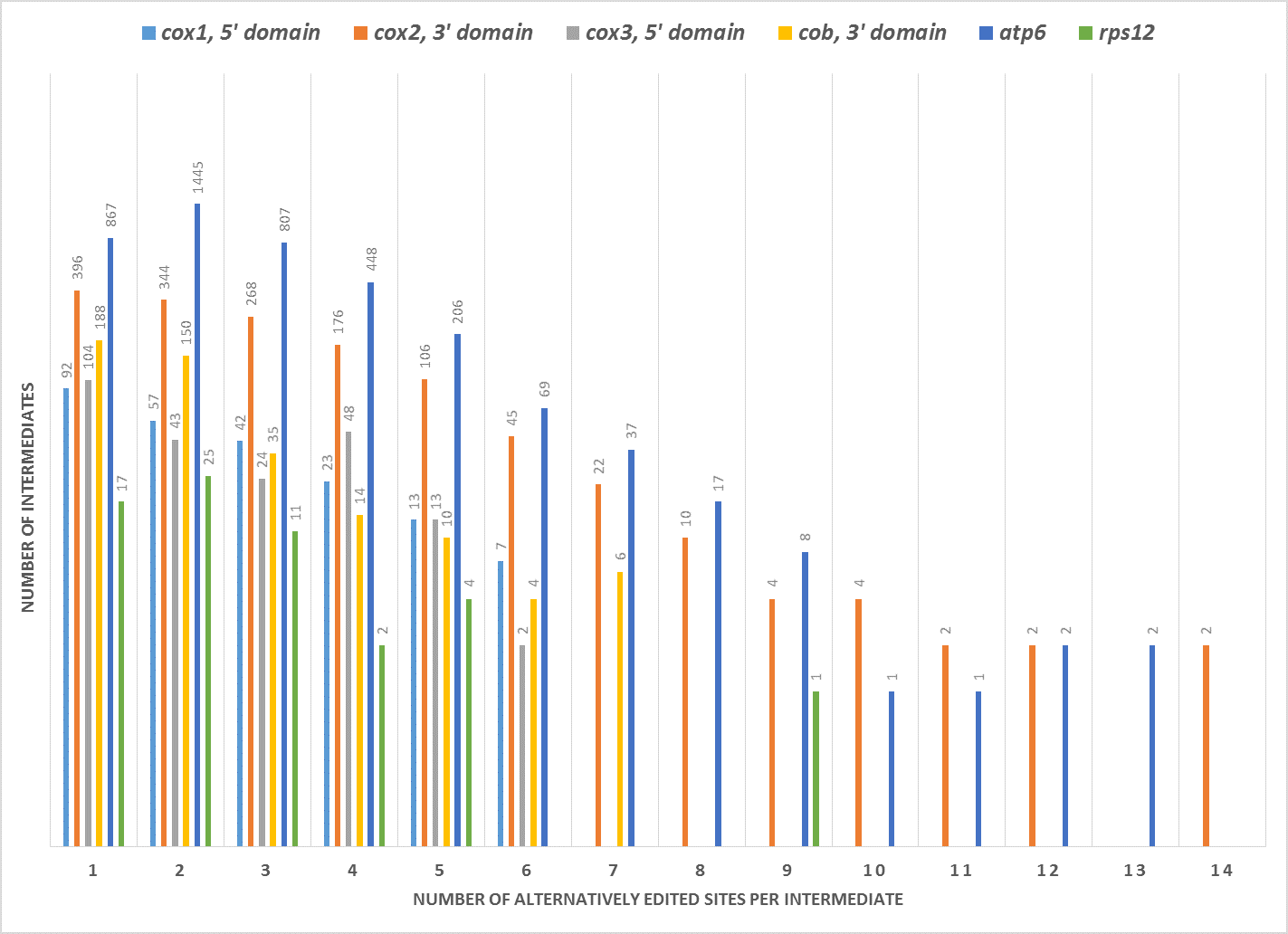

Supplement: File S5 — Alternative editing quantification. Pie charts illustrating counts of reads matching the main editing pathway, the pre-edited sequence, and alternatively edited reads in Perkinsela strains CCAP1560/4 and GillNOR1/I (A). Alternative editing intermediates in Perkinsela sp. Mitochondria, sorted according to their rounded support values (B) and number of editing sites (C) (x-axis) are shown. Read counts in logarithmic scale (y-axis) are given separately for each model edited domain (strain CCAP1560/4 for cox1, cox3, atp6; strain GillNOR1/I for cox2, cob, rps12). Download [file mbo005152537s5.docx]
