# Supplementary material for: Gene Loss and Error-Prone RNA Editing in the Mitochondrion of Perkinsela, an Endosymbiotic Kinetoplastid
Source: mBio. 2015 Dec 1;6(6):e01498-15. doi: 10.1128/mBio.01498-15 (PMC4669381; doi:10.1128/mBio.01498-15)

***cox2***, 3′ edited domain (partial), ~200 non-redundant reads with highest support values and reads with >10 alternatively edited sites (at the bottom), GillNOR1/I


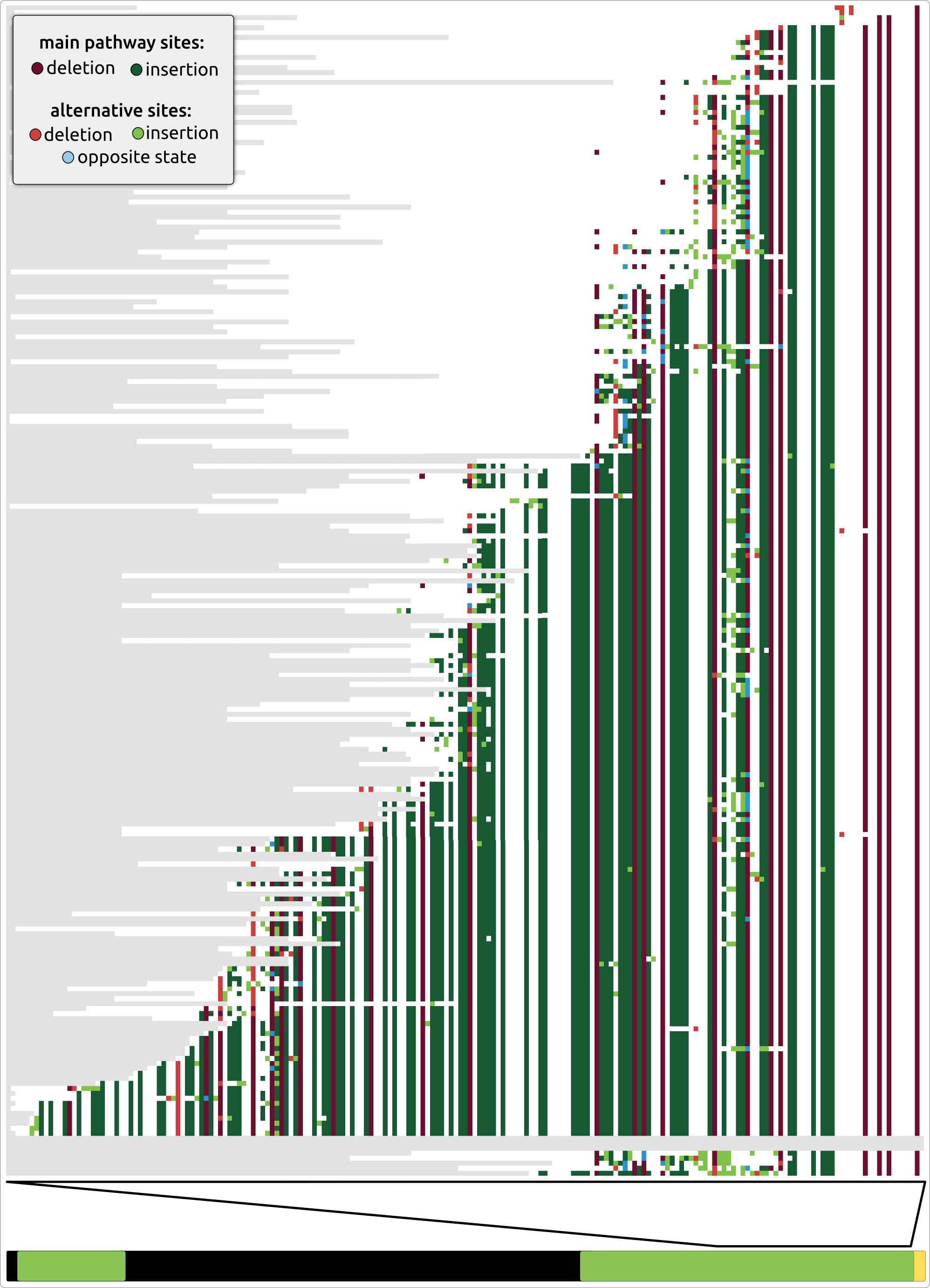


***cox1***, 5′ edited domain (full), all non-redundant reads, CCAP1560/4

**
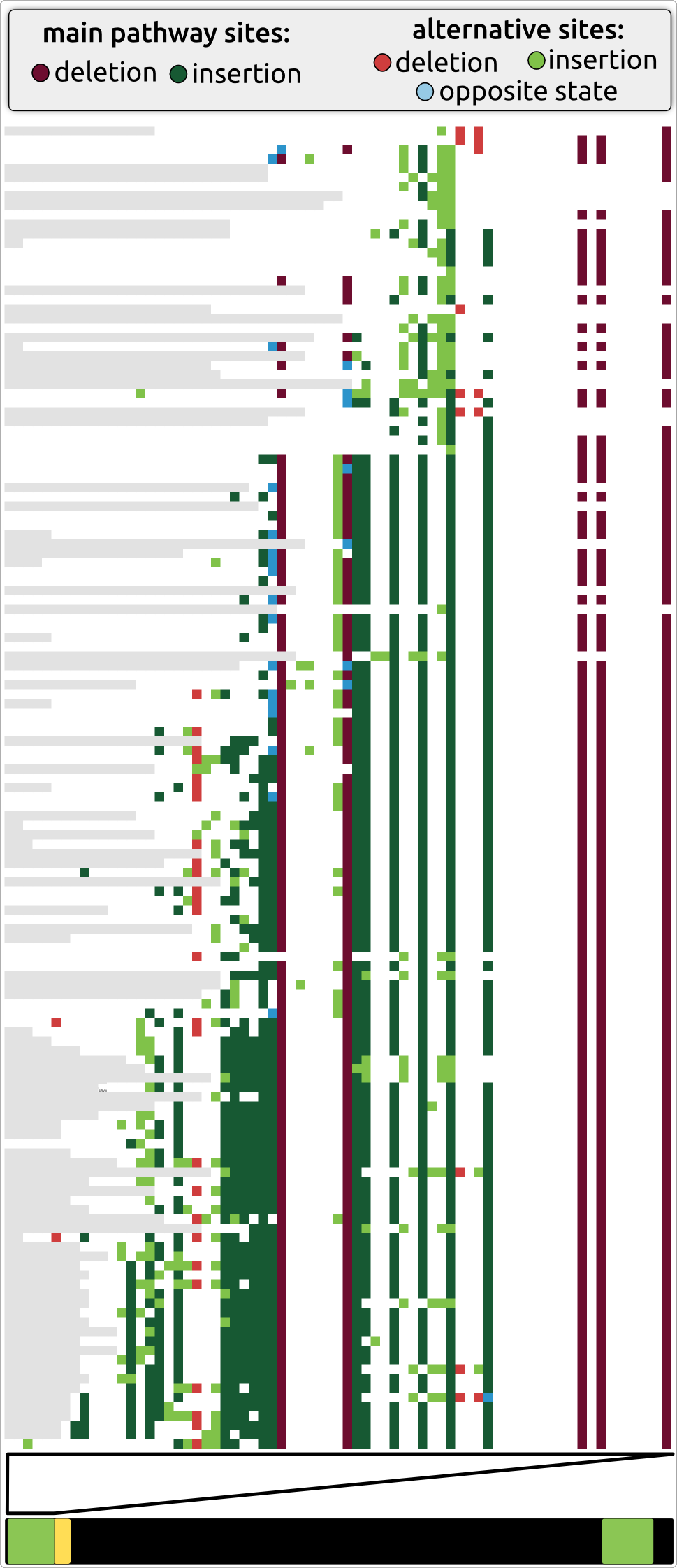
**

***cox3***, 5′ edited domain (full), all non-redundant reads, CCAP1560/4


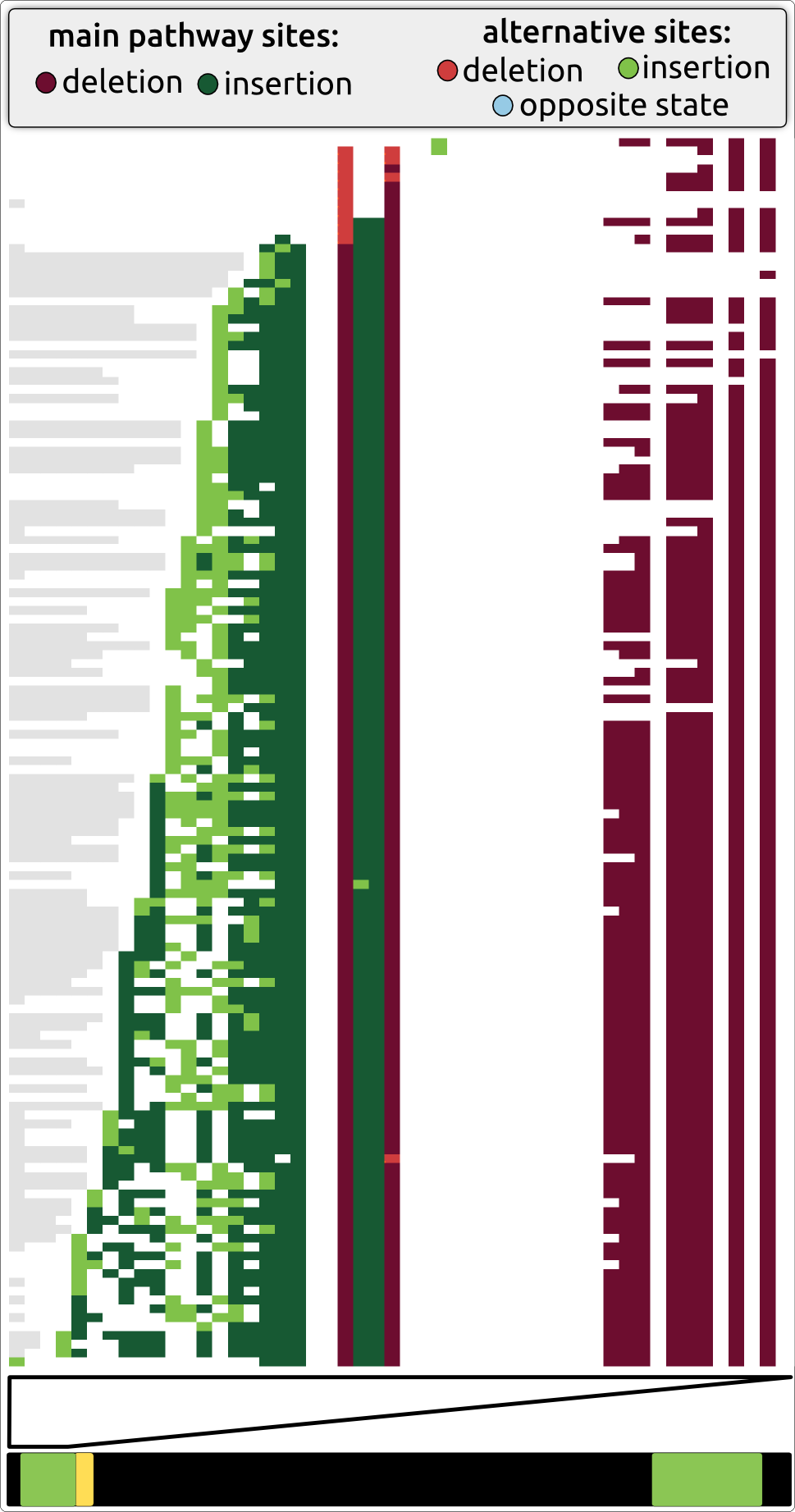


***cob***, 3′ edited domain (partial), all non-redundant reads, GillNOR1/I

**
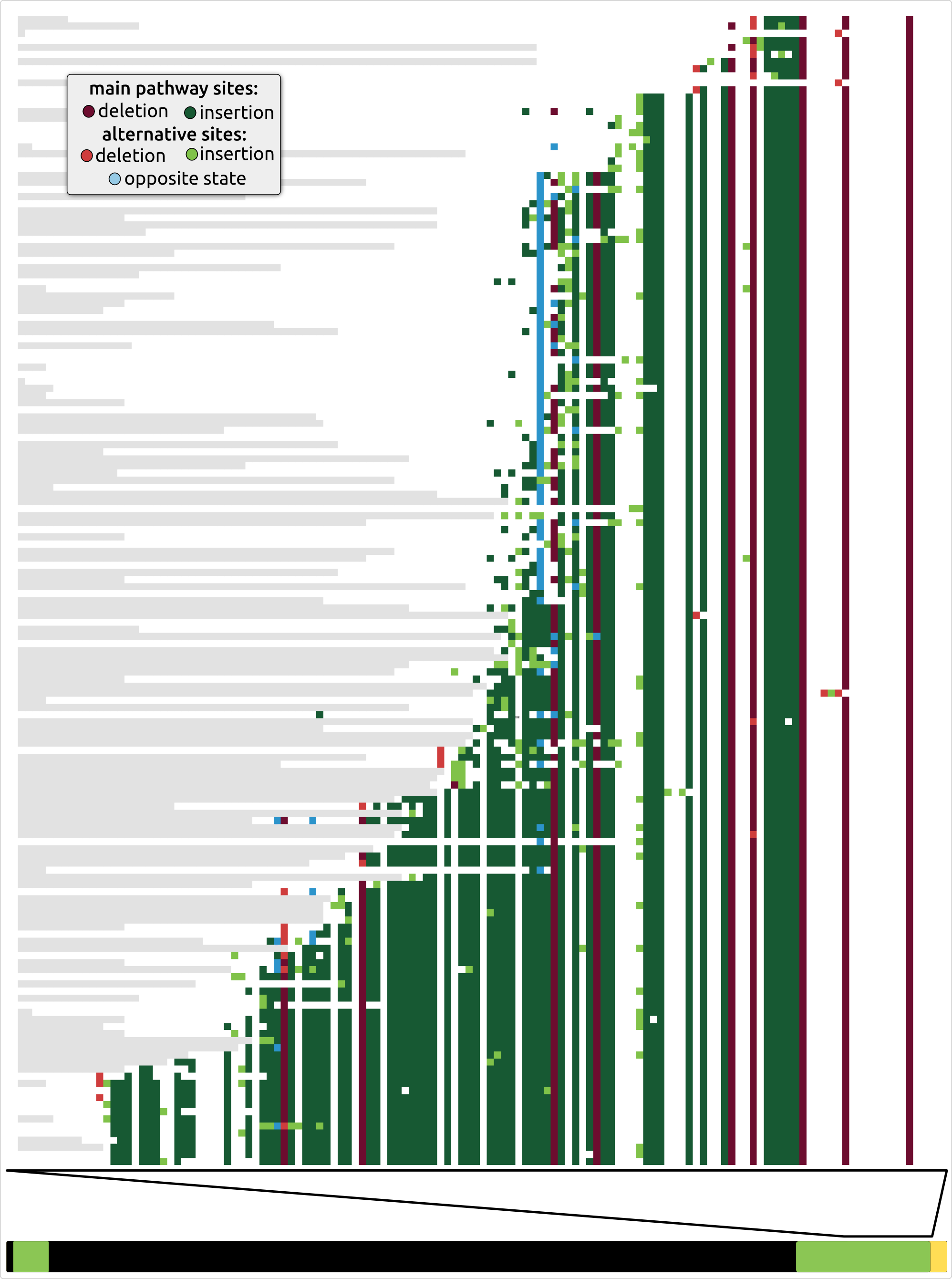
**

***atp6*** (partial), non-redundant reads with highest support values, CCAP1560/4

**
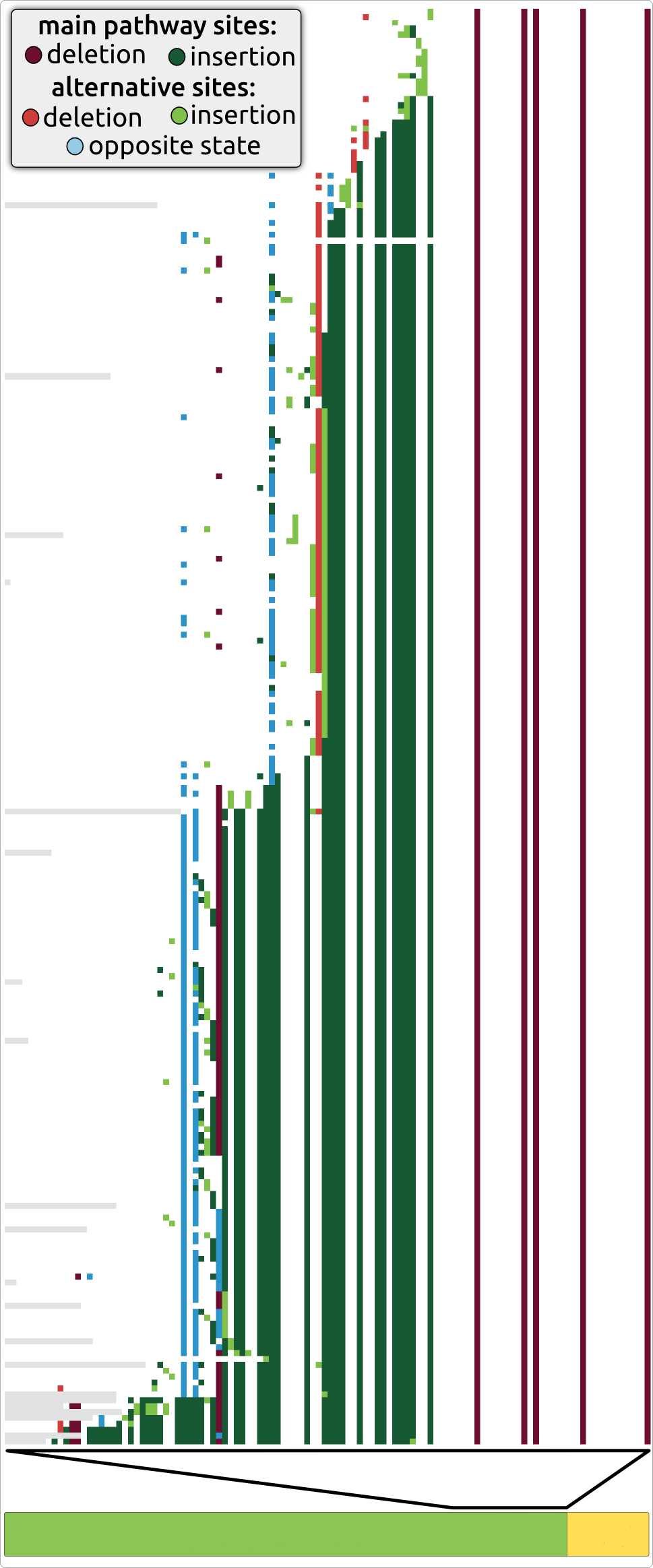
**

***rps12*** (partial), all non-redundant reads, GillNOR1/I


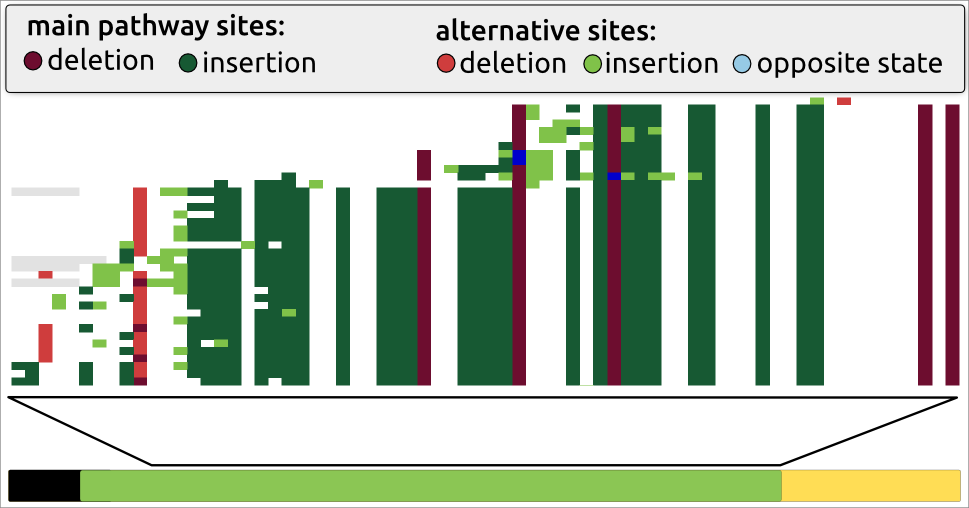

Supplement: File S1 — Alternatively edited and “misedited” products for cox1, cox2, cox3, cob, atp6, and rps12. The figure represents an output of T-aligner, illustrating non-redundant alternatively edited reads (also termed "editing intermediates" in this paper to distinguish them from reads in the raw dataset). Each column represents an editing site irrespective of the U-indel length observed; each line represents a read with its sequence in white. U-insertion and U-deletion sites identical to those in the main edited product are shown in dark green and dark red, respectively. A site is considered alternatively edited if any of the following conditions are met: (i) a site is never-edited in the main editing product; (ii) U indel is longer than in the main product; (iii) U insertion occurs instead of deletion in the main product or vice versa. Alternatively edited sites are shown in bright green and orange for U insertions and U deletions, respectively, and sites with the editing direction reversed (group iii) are denoted in blue. The transcript is schematically shown at the bottom, with edited domains in green and the seed used by T-aligner in yellow. Download [file mbo005152537s1.docx]
