# Supplementary material for: Gene Loss and Error-Prone RNA Editing in the Mitochondrion of Perkinsela, an Endosymbiotic Kinetoplastid
Source: mBio. 2015 Dec 1;6(6):e01498-15. doi: 10.1128/mBio.01498-15 (PMC4669381; doi:10.1128/mBio.01498-15)

Genomic and transcriptomic NGS reads of *Perkinsela* CCAP1560/4 and GillNOR1/I strains.

| **strain** | **molecule** | **platform** | **type** | **insert size, nt** | **status** | **reads** | **av. length, nt** | **av. coverage, *cox2* contig^a^** |
| --- | --- | --- | --- | --- | --- | --- | --- | --- |
| **GillNOR1/I** | **DNA** | Illumina MiSeq, 250 bp reads | paired-end | 300-400 | raw | 2.20×10^7^ | 250 |  |
|  |  |  |  |  | trimmed | 1.51×10^7^ | 184 | 5,759 |
|  | **RNA** | Illumina MiSeq, 250 bp reads | polyA, paired-end, strand-specific | 130-380 | trimmed | 3.90×10^7^ | 179 |  |
|  |  |  |  |  | merged | 1.72×10^7^ | 192 | 1,841 |
|  |  |  | total, paired-end, strand-specific | 160-330 | trimmed | 3.80×10^7^ | 185 |  |
|  |  |  |  |  | merged | 1.75×10^7^ | 196 | 1.4 |
| **CCAP1560/4** | **DNA** | 454 GS FLX+ | single | N/A | trimmed | 1.02×10^6^ | 443 | 108 |
|  |  | Illumina HiSeq, 110bp reads | paired-end | 290-490 | raw | 5.98×10^7^ | 110 |  |
|  |  |  |  |  | trimmed | 4.54×10^7^ | 107 | 1,058 |
|  |  |  | mate pair | 3000-5000 | raw | 4.84×10^8^ | 101 |  |
|  |  |  |  |  | trimmed | 4.20×10^8^ | 99 | 15,831 |
|  | **RNA** | Illumina HiSeq, 100 bp reads | polyA, paired-end, non-strand-specific | 50-250 | raw | 2.76×10^8^ | 100 |  |
|  |  |  |  |  | trimmed | 2.65×10^8^ | 97 |  |
|  |  |  |  |  | merged | 6.60×10^7^ | 149 | 1,240 |

^a^ based on read mapping with CLC Genomics Workbench v.6.5 (read length fraction mapped > 0.9, percent identity within the mapped region > 0.7)
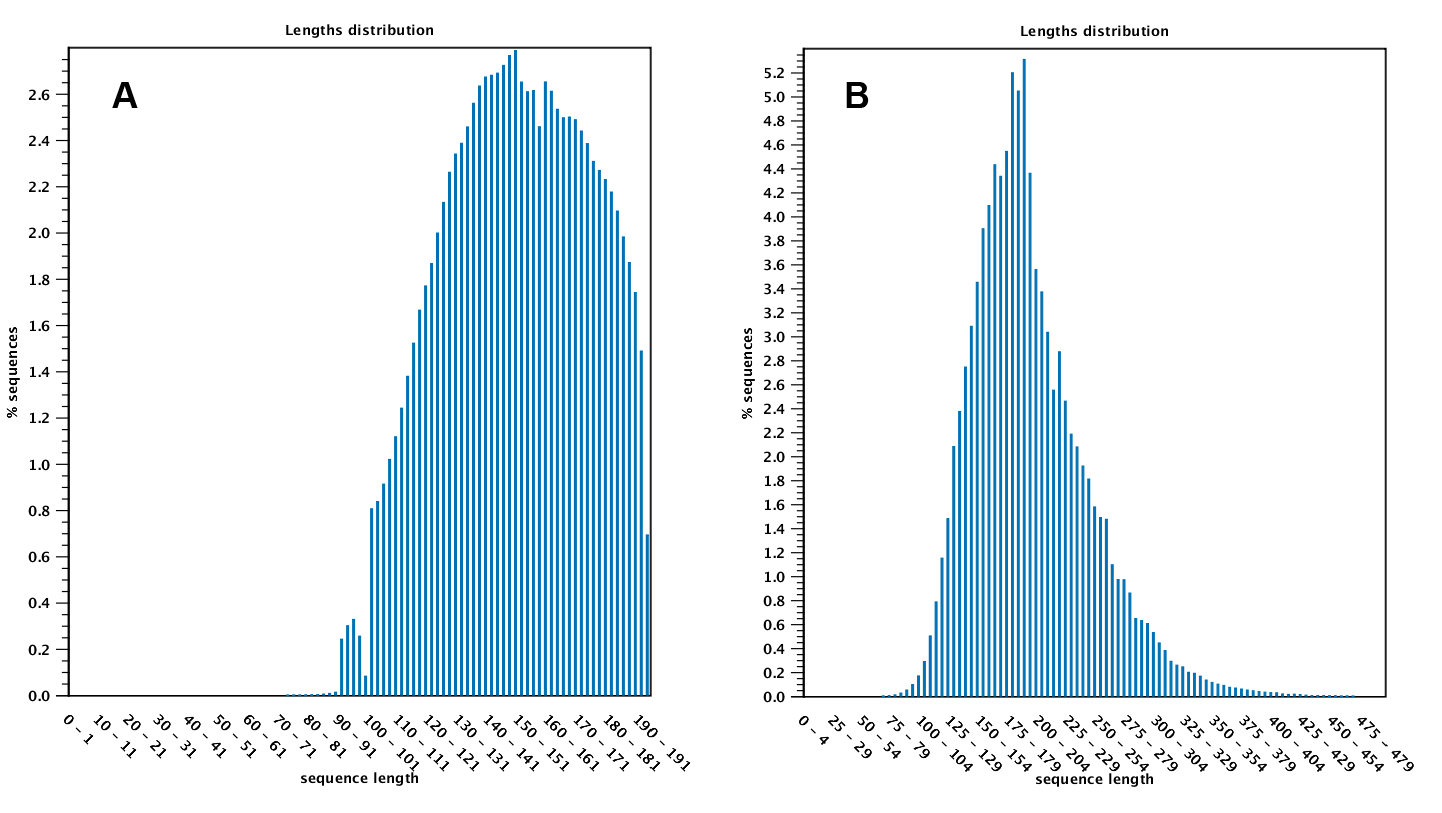

Supplement: File S2 — Genomic and transcriptomic NGS reads. Paired transcriptomic reads were merged with the CLC Genomics Workbench v.6.5 prior to mapping. Merged read length distribution is shown for the Perkinsela CCAP1560/4 (A) and GillNOR1/I strains (B). The abrupt edges of the distribution in panel A are due to 100 bp and shorter trimmed reads which produce merged reads of 190 bp or shorter, if a minimum overlap of 10 bp is required. Download [file mbo005152537s2.docx]
