# Supplementary material for: Gene Loss and Error-Prone RNA Editing in the Mitochondrion of Perkinsela, an Endosymbiotic Kinetoplastid
Source: mBio. 2015 Dec 1;6(6):e01498-15. doi: 10.1128/mBio.01498-15 (PMC4669381; doi:10.1128/mBio.01498-15)
Supplement: File S3 — Guide RNA gene candidates revealed in the genome assembly of Perkinsela sp. CCAP1560/4. Aligned stretches of gRNA and mRNA (at least 25 bp in length) are shown in uppercase, and their flanks are shown in lowercase. Contig IDs and start coordinates are shown, along with a number of matching transcriptomic reads (support). The 5′ AC-rich region of gRNAs is underlined. Download [file mbo005152537s3.docx]

Potential gRNA for atp6: contig11121 (at 457), support=1

mRNA: 5'-atttgtttttgttacGTTTTTTTGTTGTTTTCTTGTTGTTgttgcgtttgttgag-3'

mRNA: 3'-gtaacaaaagagtaaCAAAAGAGTAACAAAAGAGTAACAAaagaacatccttcac-5'

Potential gRNA for atp6: contig11121 (at 468), support=1

mRNA: 5'-atttgtttttgttacGTTTTTTTGTTGTTTTCTTGTTGTTgttgcgtttgttgag-3'

mRNA: 3'-gtaacaaaagagtaaCAAAAGAGTAACAAAAGAGTAACAAaagagtaacaaaaga-5'

Potential gRNA for atp6: contig11121 (at 479), support=1

mRNA: 5'-atttgtttttgttacGTTTTTTTGTTGTTTTCTTGTTGTTgttgcgtttgttgag-3'

mRNA: 3'-acaacaaaagagtaaCAAAAGAGTAACAAAAGAGTAACAAaagagtaacaaaaga-5'

Potential gRNA for atp6: contig11121 (at 490), support=1

mRNA: 5'-atttgtttttgttacGTTTTTTTGTTGTTTTCTTGTTGTTgttgcgtttgttgag-3'

mRNA: 3'-accaacaaaagacaaCAAAAGAGTAACAAAAGAGTAACAAaagagtaacaaaaga-5'

Potential gRNA for atp6: contig42579 (at 56), support=1

mRNA: 5'-atttgtttttgttacGTTTTTTTGTTGTTTTCTTGTTGTTgttgcgtttgttgag-3'

mRNA: 3'-caattaaaagaacaaTAAAAGAACAATAAAAGAACGATAAaagaacatttagaca-5'

Potential gRNA for atp6: contig42579 (at 67), support=1

mRNA: 5'-atttgtttttgttacGTTTTTTTGTTGTTTTCTTGTTGTTgttgcgtttgttgag-3'

mRNA: 3'-tgctgctcgtccaatTAAAAGAACAATAAAAGAACAATAAaagaacgataaaaga-5'

Potential gRNA for atp6: contig08597 (at 1151), support=5

mRNA: 5'-tttgtttttgttacgTTTTTTTGTTGTTTTCTTGTTGTTGttgcgtttgttgagt-3'

mRNA: 3'-acaagaaaatgaggaGAAGGAACAACAAGAGAACAATAACaaccacaacaacaag-5'

--------------

Potential gRNA for atp6: contig22215 (at 71), support=1

mRNA: 5'-tttgtttttgttacgTTTTTTTGTTGTTTTCTTGTTGTTGttgcgtttgttgagt-3'

mRNA: 3'-aactttctctccaaaAAAAGAATAACAAGAGAACAACAACaacaacaacaacaac-5'

---------------

Potential gRNA for atp6: contig08597 (at 1150), support=5

mRNA: 5'-ttgtttttgttacgtTTTTTTGTTGTTTTCTTGTTGTTGTtgcgtttgttgagtt-3'

mRNA: 3'-caagaaaatgaggagAAGGAACAACAAGAGAACAATAACAaccacaacaacaaga-5'

-------------

Potential gRNA for atp6: contig22215 (at 70), support=1

mRNA: 5'-ttgtttttgttacgtTTTTTTGTTGTTTTCTTGTTGTTGTtgcgtttgttgagtt-3'

mRNA: 3'-actttctctccaaaaAAAGAATAACAAGAGAACAACAACAacaacaacaacaaca-5'

---------------

Potential gRNA for atp6: contig16894 (at 169) (revcom), support=3

mRNA: 5'-ttgtttttgttacgtTTTTTTGTTGTTTTCTTGTTGTTGTtgcgtttgttgagtt-3'

mRNA: 3'-aacgacaacaacaacGAAAAACAACAGAAGAACAATAACActaaagaaaaaaaga-5'

-----------

Potential gRNA for atp6: contig08597 (at 1149), support=5

mRNA: 5'-tgtttttgttacgttTTTTTGTTGTTTTCTTGTTGTTGTTgcgtttgttgagttt-3'

mRNA: 3'-aagaaaatgaggagaAGGAACAACAAGAGAACAATAACAAccacaacaacaagaa-5'

------------

Potential gRNA for atp6: contig22215 (at 69), support=1

mRNA: 5'-tgtttttgttacgttTTTTTGTTGTTTTCTTGTTGTTGTTgcgtttgttgagttt-3'

mRNA: 3'-ctttctctccaaaaaAAGAATAACAAGAGAACAACAACAAcaacaacaacaacaa-5'

---------------

Potential gRNA for atp6: contig08597 (at 1148), support=5

mRNA: 5'-gtttttgttacgtttTTTTGTTGTTTTCTTGTTGTTGTTGcgtttgttgagtttg-3'

mRNA: 3'-agaaaatgaggagaaGGAACAACAAGAGAACAATAACAACcacaacaacaagaag-5'

-----------

Potential gRNA for atp6: contig22215 (at 68), support=1

mRNA: 5'-gtttttgttacgtttTTTTGTTGTTTTCTTGTTGTTGTTGcgtttgttgagtttg-3'

mRNA: 3'-tttctctccaaaaaaAGAATAACAAGAGAACAACAACAACaacaacaacaacaac-5'

---------------

Potential gRNA for atp6: contig17031 (at 203) (revcom), support=4

mRNA: 5'-gtattttttatttgaTGTTTTGTTGTTTGTTTCTTTTTTGgttggggtttataat-3'

mRNA: 3'-tatgaatatgtatgcATGAAACAACAAACAAAGAAAAGACtcgcgttacgttgtc-5'

Potential gRNA for atp6: contig17031 (at 202) (revcom), support=4

mRNA: 5'-tattttttatttgatGTTTTGTTGTTTGTTTCTTTTTTGGttggggtttataatt-3'

mRNA: 3'-atgaatatgtatgcaTGAAACAACAAACAAAGAAAAGACTcgcgttacgttgtcg-5'

Potential gRNA for cox1: contig56332 (at 86), support=18

mRNA: 5'-ggttgtttatttatgGTGTGTTTTATTGTAGTTGTTGAGTtgggttttttttatt-3'

mRNA: 3'-tcctatgaatatgtaTGCATGAAATAACATCAACAACTCAgactcgcgttacgtt-5'

Potential gRNA for cox1: contig12621 (at 725) (revcom), support=18

mRNA: 5'-ggttgtttatttatgGTGTGTTTTATTGTAGTTGTTGAGTtgggttttttttatt-3'

mRNA: 3'-tcctatgaatatgtaTGCATGAAATAACATCAACAACTCAgactcgcgttacgtt-5'

Potential gRNA for cox1: contig56332 (at 85), support=13

mRNA: 5'-gttgtttatttatggTGTGTTTTATTGTAGTTGTTGAGTTgggttttttttattg-3'

mRNA: 3'-cctatgaatatgtatGCATGAAATAACATCAACAACTCAGactcgcgttacgttg-5'

Potential gRNA for cox1: contig12621 (at 724) (revcom), support=13

mRNA: 5'-gttgtttatttatggTGTGTTTTATTGTAGTTGTTGAGTTgggttttttttattg-3'

mRNA: 3'-cctatgaatatgtatGCATGAAATAACATCAACAACTCAGactcgcgttacgttg-5'

Potential gRNA for cox2: contig23071 (at 328), support=1

mRNA: 5'-gtttttgtttggttgTTTGTTTTGTTTTGGGTTGGATTGTtttatgtttgtattt-3'

mRNA: 3'-aaaaaacaaaacaaaAAACAAAACAAAACCTAACCTAACAacgaaaatgtctggc-5'

Potential gRNA for cox2: contig23071 (at 327), support=1

mRNA: 5'-tttttgtttggttgtTTGTTTTGTTTTGGGTTGGATTGTTttatgtttgtatttt-3'

mRNA: 3'-aaaaacaaaacaaaaAACAAAACAAAACCTAACCTAACAAcgaaaatgtctggcc-5'

Potential gRNA for cox2: contig46554 (at 170), support=3

mRNA: 5'-gtttggttgtttgttTTGTTTTGGGTTGGATTGTTTTATGtttgtattttgtttt-3'

mRNA: 3'-tttttttaactaattAACAAAATTTAACTTAACAAAATACaaaatttttttatta-5'

Potential gRNA for cox2: contig46554 (at 169), support=3

mRNA: 5'-tttggttgtttgtttTGTTTTGGGTTGGATTGTTTTATGTttgtattttgtttta-3'

mRNA: 3'-ttttttaactaattaACAAAATTTAACTTAACAAAATACAaaatttttttattat-5'

Potential gRNA for cox2: contig46554 (at 168), support=3

mRNA: 5'-ttggttgtttgttttGTTTTGGGTTGGATTGTTTTATGTTtgtattttgttttag-3'

mRNA: 3'-tttttaactaattaaCAAAATTTAACTTAACAAAATACAAaatttttttattatt-5'

Potential gRNA for cox2: contig46554 (at 167), support=3

mRNA: 5'-tggttgtttgttttgTTTTGGGTTGGATTGTTTTATGTTTgtattttgttttagt-3'

mRNA: 3'-ttttaactaattaacAAAATTTAACTTAACAAAATACAAAatttttttattatta-5'

Potential gRNA for cox2: contig06030 (at 538), support=1

mRNA: 5'-ttttgttttgggttgGATTGTTTTATGTTTGTATTTTGTTttagtttttttttga-3'

mRNA: 3'-taaatgtttttatgtTTAATAAAATATAAATGTAAAACAAgttttttgcttggga-5'

Potential gRNA for cox2: contig06030 (at 537), support=1

mRNA: 5'-tttgttttgggttggATTGTTTTATGTTTGTATTTTGTTTtagtttttttttgat-3'

mRNA: 3'-aaatgtttttatgttTAATAAAATATAAATGTAAAACAAGttttttgcttgggag-5'

Potential gRNA for cox2: contig11508 (at 330), support=14

mRNA: 5'-tttgtattttgttttAGTTTTTTTTTGATCTGTTGGTTTTgtatttggttctttg-3'

mRNA: 3'-cgtcgggaaaaaaccTCAAGAGAAAGTTAGACAACTAAAAcgtgactcctgtcac-5'

Potential gRNA for cox2: contig14393 (at 607), support=14

mRNA: 5'-tttgtattttgttttAGTTTTTTTTTGATCTGTTGGTTTTgtatttggttctttg-3'

mRNA: 3'-ccgccgggaaaaaccTCAAGAGAAAGTTAGACAACTAAAAcgtgactcctgtcac-5'

Potential gRNA for cox2: contig20639 (at 319), support=14

mRNA: 5'-tttgtattttgttttAGTTTTTTTTTGATCTGTTGGTTTTgtatttggttctttg-3'

mRNA: 3'-cgccgggaaaaaaccTCAAGAGAAAGTTAGACAACTAAAAcgtgactcctgtcac-5'

Potential gRNA for cox2: contig33597 (at 59), support=14

mRNA: 5'-tttgtattttgttttAGTTTTTTTTTGATCTGTTGGTTTTgtatttggttctttg-3'

mRNA: 3'-cgccgggaaaaaaccTCAAGAGAAAGTTAGACAACTAAAAcgtgactcctgtcac-5'

Potential gRNA for cox2: contig33605 (at 168), support=14

mRNA: 5'-tttgtattttgttttAGTTTTTTTTTGATCTGTTGGTTTTgtatttggttctttg-3'

mRNA: 3'-ccgtcgggaaaaaccTCAAGAGAAAGTTAGACAACTAAAAcgtgactcctgtcac-5'

Potential gRNA for cox2: contig10544 (at 414) (revcom), support=14

mRNA: 5'-tttgtattttgttttAGTTTTTTTTTGATCTGTTGGTTTTgtatttggttctttg-3'

mRNA: 3'-cgccgggaaaaaaccTCAAGAGAAAGTTAGACAACTAAAAcgtgactcctgtcac-5'

Potential gRNA for cox2: contig11909 (at 893) (revcom), support=14

mRNA: 5'-tttgtattttgttttAGTTTTTTTTTGATCTGTTGGTTTTgtatttggttctttg-3'

mRNA: 3'-cgccgggaaaaaaccTCAAGAGAAAGTTAGACAACTAAAAcgtgactcctgtcac-5'

Potential gRNA for cox2: contig16686 (at 492) (revcom), support=14

mRNA: 5'-tttgtattttgttttAGTTTTTTTTTGATCTGTTGGTTTTgtatttggttctttg-3'

mRNA: 3'-gccgggaaaaaaaccTCAAGAGAAAGTTAGACAACTAAAAcgtgactcctgtcac-5'

Potential gRNA for cox2: contig17676 (at 454) (revcom), support=14

mRNA: 5'-tttgtattttgttttAGTTTTTTTTTGATCTGTTGGTTTTgtatttggttctttg-3'

mRNA: 3'-cgccgggaaaaaaccTCAAGAGAAAGTTAGACAACTAAAAcgtgactcctgtcac-5'

Potential gRNA for cox2: contig23913 (at 297) (revcom), support=14

mRNA: 5'-tttgtattttgttttAGTTTTTTTTTGATCTGTTGGTTTTgtatttggttctttg-3'

mRNA: 3'-cgccgggaaaaaaccTCAAGAGAAAGTTAGACAACTAAAAcgtgactcctgtcac-5'

Potential gRNA for cox2: contig24965 (at 332) (revcom), support=14

mRNA: 5'-tttgtattttgttttAGTTTTTTTTTGATCTGTTGGTTTTgtatttggttctttg-3'

mRNA: 3'-gccgggaaaaaaaccTCAAGAGAAAGTTAGACAACTAAAAcgtgactcctgtcac-5'

Potential gRNA for cox2: contig25986 (at 336) (revcom), support=14

mRNA: 5'-tttgtattttgttttAGTTTTTTTTTGATCTGTTGGTTTTgtatttggttctttg-3'

mRNA: 3'-cgccgggaaaaaaccTCAAGAGAAAGTTAGACAACTAAAAcgtgactcctgtcac-5'

Potential gRNA for cox2: contig39618 (at 129) (revcom), support=14

mRNA: 5'-tttgtattttgttttAGTTTTTTTTTGATCTGTTGGTTTTgtatttggttctttg-3'

mRNA: 3'-cgccgggaaaaaaccTCAAGAGAAAGTTAGACAACTAAAAcgtgactcctgtcac-5'

Potential gRNA for cox2: contig11508 (at 329), support=15

mRNA: 5'-ttgtattttgttttaGTTTTTTTTTGATCTGTTGGTTTTGtatttggttctttgt-3'

mRNA: 3'-gtcgggaaaaaacctCAAGAGAAAGTTAGACAACTAAAACgtgactcctgtcacc-5'

Potential gRNA for cox2: contig14393 (at 606), support=15

mRNA: 5'-ttgtattttgttttaGTTTTTTTTTGATCTGTTGGTTTTGtatttggttctttgt-3'

mRNA: 3'-cgccgggaaaaacctCAAGAGAAAGTTAGACAACTAAAACgtgactcctgtcacc-5'

Potential gRNA for cox2: contig20639 (at 318), support=15

mRNA: 5'-ttgtattttgttttaGTTTTTTTTTGATCTGTTGGTTTTGtatttggttctttgt-3'

mRNA: 3'-gccgggaaaaaacctCAAGAGAAAGTTAGACAACTAAAACgtgactcctgtcacc-5'

Potential gRNA for cox2: contig33597 (at 58), support=15

mRNA: 5'-ttgtattttgttttaGTTTTTTTTTGATCTGTTGGTTTTGtatttggttctttgt-3'

mRNA: 3'-gccgggaaaaaacctCAAGAGAAAGTTAGACAACTAAAACgtgactcctgtcacc-5'

Potential gRNA for cox2: contig33605 (at 167), support=15

mRNA: 5'-ttgtattttgttttaGTTTTTTTTTGATCTGTTGGTTTTGtatttggttctttgt-3'

mRNA: 3'-cgtcgggaaaaacctCAAGAGAAAGTTAGACAACTAAAACgtgactcctgtcacc-5'

Potential gRNA for cox2: contig10544 (at 413) (revcom), support=15

mRNA: 5'-ttgtattttgttttaGTTTTTTTTTGATCTGTTGGTTTTGtatttggttctttgt-3'

mRNA: 3'-gccgggaaaaaacctCAAGAGAAAGTTAGACAACTAAAACgtgactcctgtcacc-5'

Potential gRNA for cox2: contig11909 (at 892) (revcom), support=15

mRNA: 5'-ttgtattttgttttaGTTTTTTTTTGATCTGTTGGTTTTGtatttggttctttgt-3'

mRNA: 3'-gccgggaaaaaacctCAAGAGAAAGTTAGACAACTAAAACgtgactcctgtcacc-5'

Potential gRNA for cox2: contig16686 (at 491) (revcom), support=15

mRNA: 5'-ttgtattttgttttaGTTTTTTTTTGATCTGTTGGTTTTGtatttggttctttgt-3'

mRNA: 3'-ccgggaaaaaaacctCAAGAGAAAGTTAGACAACTAAAACgtgactcctgtcacc-5'

Potential gRNA for cox2: contig17676 (at 453) (revcom), support=15

mRNA: 5'-ttgtattttgttttaGTTTTTTTTTGATCTGTTGGTTTTGtatttggttctttgt-3'

mRNA: 3'-gccgggaaaaaacctCAAGAGAAAGTTAGACAACTAAAACgtgactcctgtcacc-5'

Potential gRNA for cox2: contig23913 (at 296) (revcom), support=15

mRNA: 5'-ttgtattttgttttaGTTTTTTTTTGATCTGTTGGTTTTGtatttggttctttgt-3'

mRNA: 3'-gccgggaaaaaacctCAAGAGAAAGTTAGACAACTAAAACgtgactcctgtcacc-5'

Potential gRNA for cox2: contig24965 (at 331) (revcom), support=15

mRNA: 5'-ttgtattttgttttaGTTTTTTTTTGATCTGTTGGTTTTGtatttggttctttgt-3'

mRNA: 3'-ccgggaaaaaaacctCAAGAGAAAGTTAGACAACTAAAACgtgactcctgtcacc-5'

Potential gRNA for cox2: contig25986 (at 335) (revcom), support=15

mRNA: 5'-ttgtattttgttttaGTTTTTTTTTGATCTGTTGGTTTTGtatttggttctttgt-3'

mRNA: 3'-gccgggaaaaaacctCAAGAGAAAGTTAGACAACTAAAACgtgactcctgtcacc-5'

Potential gRNA for cox2: contig39618 (at 128) (revcom), support=15

mRNA: 5'-ttgtattttgttttaGTTTTTTTTTGATCTGTTGGTTTTGtatttggttctttgt-3'

mRNA: 3'-gccgggaaaaaacctCAAGAGAAAGTTAGACAACTAAAACgtgactcctgtcacc-5'

Potential gRNA for cox3: contig51860 (at 125) (revcom), support=1

mRNA: 5'-ttgttatgtattctgATGTGTTGTTTTTTCTTGTTGGGTGgttatttgtttgttg-3'

mRNA: 3'-aatatgtatgcatgaTACACAACAAAAAAGAACGACTCGCgttacgttgtcggtt-5'

Potential gRNA for cyb: contig01073 (at 1718) (revcom), support=10

mRNA: 5'-tctattcccagtaaaTTTTTTGGTGTTTTGTTGTTGTTGGtttttgtttcttgtt-3'

mRNA: 3'-cccaaaaacaaaacaAAAAAACCGTAGAACAACAACAACCgtcacacaaacaaat-5'

------------

Potential gRNA for cyb: contig01073 (at 1717) (revcom), support=11

mRNA: 5'-ctattcccagtaaatTTTTTGGTGTTTTGTTGTTGTTGGTttttgtttcttgttg-3'

mRNA: 3'-ccaaaaacaaaacaaAAAAACCGTAGAACAACAACAACCGtcacacaaacaaatg-5'

-------------

Potential gRNA for cyb: contig18877 (at 274) (revcom), support=114

mRNA: 5'-ttttgttgttgttggTTTTTGTTTCTTGTTGGTCTTTTTTgctgtgttggttatc-3'

mRNA: 3'-aaccaaaactgttgaAAAAACAAAGAACAGCCAGAGAAGGaaaattttcaccaaa-5'

Potential gRNA for cyb: contig09010 (at 1256), support=3

mRNA: 5'-tggtattgtttcgttTTTTTGTTTTTTGTATTGGTTTTTAttgtttgtagttggt-3'

mRNA: 3'-ggagttccgacgcatAAAAACGAAAAACGTAATTAGAAATaaaaagtaaaaaaaa-5'

---------------

Potential gRNA for cyb: contig04349 (at 716) (revcom), support=1

mRNA: 5'-tggtattgtttcgttTTTTTGTTTTTTGTATTGGTTTTTAttgtttgtagttggt-3'

mRNA: 3'-aataaaaataaaaatAAAAATAAAAAACATGACTAAAGGTaaacatcctttaata-5'

--------

Potential gRNA for cyb: contig09010 (at 1255), support=3

mRNA: 5'-ggtattgtttcgtttTTTTGTTTTTTGTATTGGTTTTTATtgtttgtagttggtt-3'

mRNA: 3'-gagttccgacgcataAAAACGAAAAACGTAATTAGAAATAaaaagtaaaaaaaaa-5'

---------------

Potential gRNA for cyb: contig04349 (at 715) (revcom), support=1

mRNA: 5'-ggtattgtttcgtttTTTTGTTTTTTGTATTGGTTTTTATtgtttgtagttggtt-3'

mRNA: 3'-ataaaaataaaaataAAAATAAAAAACATGACTAAAGGTAaacatcctttaataa-5'

-------

Potential gRNA for cyb: contig09010 (at 1254), support=3

mRNA: 5'-gtattgtttcgttttTTTGTTTTTTGTATTGGTTTTTATTgtttgtagttggttg-3'

mRNA: 3'-agttccgacgcataaAAACGAAAAACGTAATTAGAAATAAaaagtaaaaaaaaac-5'

--------------

Potential gRNA for cyb: contig04349 (at 714) (revcom), support=1

mRNA: 5'-gtattgtttcgttttTTTGTTTTTTGTATTGGTTTTTATTgtttgtagttggttg-3'

mRNA: 3'-taaaaataaaaataaAAATAAAAAACATGACTAAAGGTAAacatcctttaataaa-5'

------
